# Yeast Ssd1 is a non-enzymatic member of the RNase II family with an alternative RNA recognition site

**DOI:** 10.1093/nar/gkab615

**Published:** 2021-07-24

**Authors:** Rosemary A Bayne, Uma Jayachandran, Aleksandra Kasprowicz, Stefan Bresson, David Tollervey, Edward W J Wallace, Atlanta G Cook

**Affiliations:** Institute of Cell Biology and SynthSys, School of Biological Sciences, University of Edinburgh, Edinburgh EH9 3BF, UK; Wellcome Centre for Cell Biology, School of Biological Sciences, University of Edinburgh, Edinburgh EH9 3BF, UK; Wellcome Centre for Cell Biology, School of Biological Sciences, University of Edinburgh, Edinburgh EH9 3BF, UK; Wellcome Centre for Cell Biology, School of Biological Sciences, University of Edinburgh, Edinburgh EH9 3BF, UK; Wellcome Centre for Cell Biology, School of Biological Sciences, University of Edinburgh, Edinburgh EH9 3BF, UK; Institute of Cell Biology and SynthSys, School of Biological Sciences, University of Edinburgh, Edinburgh EH9 3BF, UK; Wellcome Centre for Cell Biology, School of Biological Sciences, University of Edinburgh, Edinburgh EH9 3BF, UK

## Abstract

Ssd1, a conserved fungal RNA-binding protein, is important in stress responses, cell division and virulence. Ssd1 is closely related to Dis3L2 of the RNase II family of nucleases, but lacks catalytic activity and likely suppresses translation of bound mRNAs. Previous studies identified RNA motifs enriched in Ssd1-associated transcripts, yet the sequence requirements for Ssd1 binding are not defined. Here, we identify precise binding sites of Ssd1 on RNA using *in vivo* cross-linking and cDNA analysis. These sites are enriched in 5′ untranslated regions of a subset of mRNAs encoding cell wall proteins. We identified a conserved bipartite motif that binds Ssd1 with high affinity *in vitro*. Active RNase II enzymes have a characteristic, internal RNA binding path; the Ssd1 crystal structure at 1.9 Å resolution shows that remnants of regulatory sequences block this path. Instead, RNA binding activity has relocated to a conserved patch on the surface of the protein. Structure-guided mutations of this surface prevent Ssd1 from binding RNA *in vitro* and phenocopy Ssd1 deletion *in vivo*. These studies provide a new framework for understanding the function of a pleiotropic post-transcriptional regulator of gene expression and give insights into the evolution of regulatory and binding elements in the RNase II family.

## Graphical Abstract

**Graphical Abstract:**
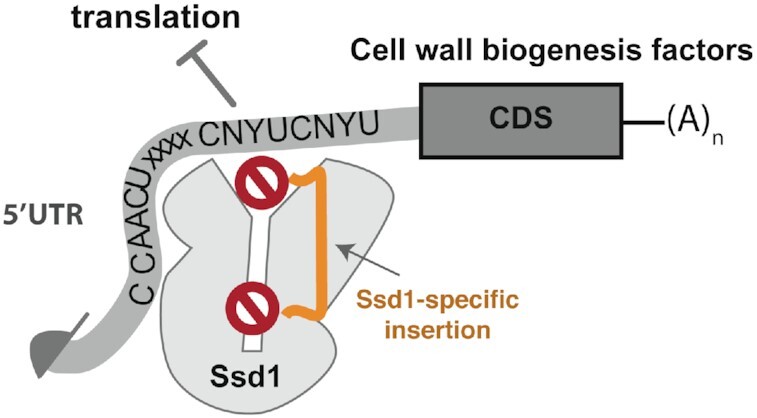
Ssd1 is a fungal RNA-binding protein that associates with transcripts required for cell wall biogenesis. Ssd1 has been proposed to block translation of associated mRNAs. We describe a specific, bipartite RNA sequence that binds to Ssd1 both *in vivo* and *in vitro*. Structural analysis of Ssd1 shows that it is a pseudonuclease where the route to the residual active site is blocked by specific loop regions. These loops are held in place by an Ssd1-specific insertion into the C-terminal domain. Instead, Ssd1 has acquired an alternative RNA binding surface.

## INTRODUCTION

Mechanisms of post-transcriptional control of gene expression by RNA-binding proteins (RBPs) include modulation of mRNA translation and decay. The RNase II/RNB family enzymes are found in all domains of life, where they play roles in RNA maturation and degradation (1). Eukaryotic DIS3 (Rrp44) and Dis3L2 are RNase II family 3′–5′ exonucleases. DIS3 or Rrp44 (for human and yeast orthologues, respectively) is the essential nuclease associated with the eukaryotic exosome complex that processes and/or turns over the majority of cellular RNAs (2). Dis3L2 is a related nuclease that is specific for RNA substrates with an oligouridine 3′ tail (3). However, some RNase II family proteins are pseudonucleases with regulatory roles in RNA metabolism, rather than active enzymes. These include the fungal Ssd1 family that is closely related to Dis3L2 (4). *Saccharomyces cerevisiae* Ssd1 binds RNA, but does not have detectable exonuclease activity (5).

Ssd1 was initially identified in *S. cerevisiae* as a genetic suppressor of mutations in the Sit4 protein phosphatase (6). *SSD1* alleles interact genetically with mutations in a number of other pathways, while loss of Ssd1 allows wild yeast variants to tolerate aneuploidy by preventing proteotoxic stress (7–11). Ssd1 homologues are important for virulence in a variety of fungal pathogens of both plants and humans (12–14). However, the molecular basis for its role in virulence is not well understood.

**Figure 1.**
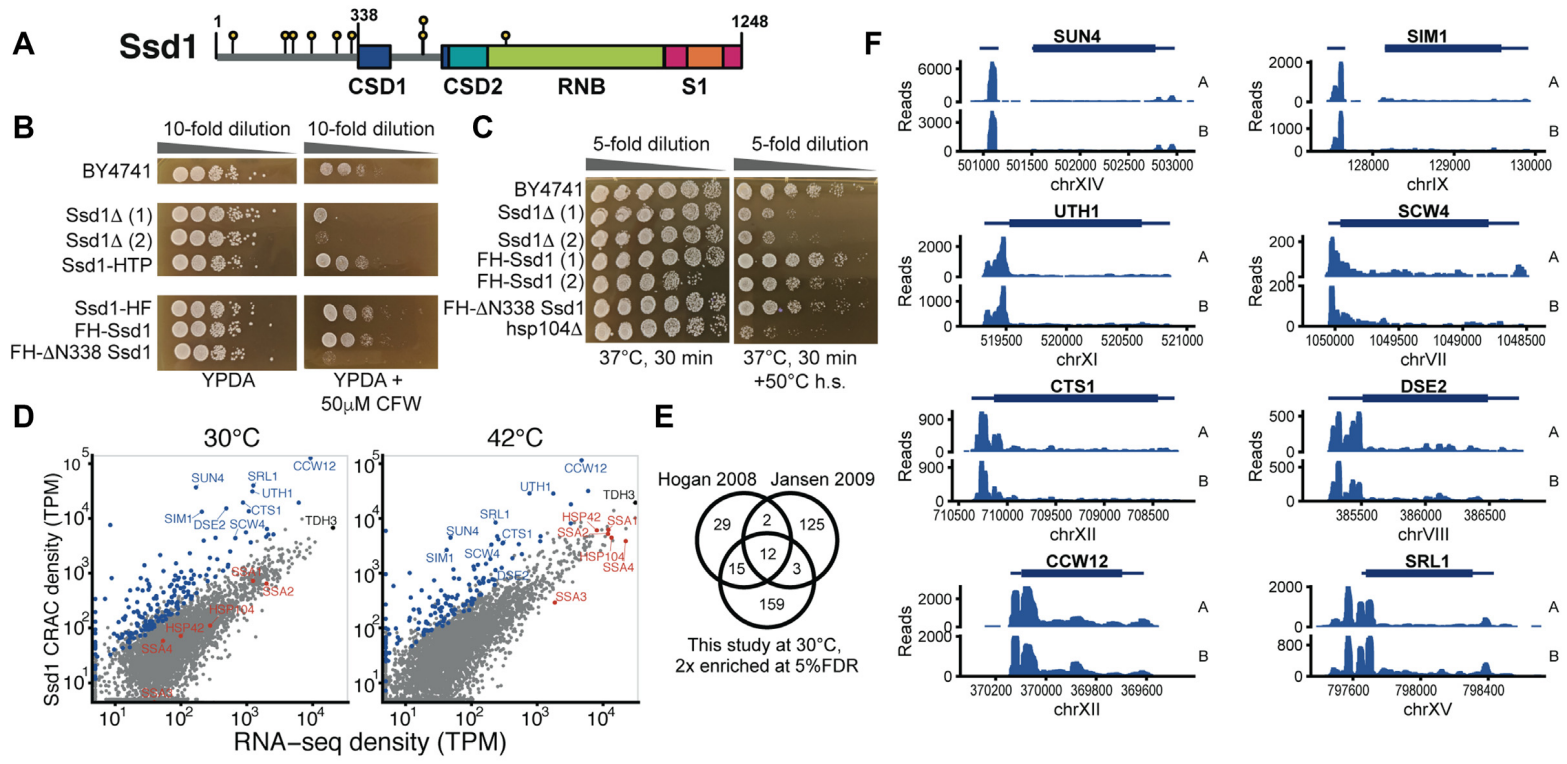
Ssd1 binds to 5′ untranslated regions (5′UTRs) of its target transcripts *in vivo*. (**A**) Domain overview of Ssd1. Boxes indicate folded domains with separating grey lines indicating natively unstructured regions; yellow lollipops indicate phosphorylation sites of Ssd1. (**B**) Wild-type and Ssd1 mutant yeast strains grown at 30°C on YPDA [yeast extract/peptone/dextrose (glucose)/adenine] without or with 50 μM calcofluor white (CFW). (**C**) Wild-type and Ssd1 or Hsp104 mutant strains grown overnight at 30°C and then incubated either at 37°C for 30 min or using an induced thermotolerance protocol. (**D**) Ssd1-bound CRAC (cross-linking and analysis of cDNAs) read density compared to RNA-seq reads in transcripts per million (TPM, mean over two biological replicates), aligned to full-length transcripts including annotated UTRs. Selected Ssd1 targets are highlighted in blue and selected heat-induced transcripts in red. (**E**) Comparison of Ssd1-bound mRNAs reported by CRAC analysis with previous RNA immunoprecipitation and microarray studies that were also conducted in rich media at 30°C. We conservatively report transcripts that are 4-fold enriched in Ssd1 CRAC reads compared to RNA-seq, and with at least 20 TPM in the RNA-seq data. (**F**) Unnormalized CRAC read counts (pileups) on selected Ssd1-bound transcripts from two biological replicates at 30°C, aligned to the yeast genome, with 5′UTRs oriented on the left.

*Saccharomyces cerevisiae* Ssd1 has a mainly cytoplasmic localization, moving to the yeast bud and bud neck during mitosis (15). Ssd1 localization matches that of Cbk1 kinase, which binds and phosphorylates a natively unstructured N-terminal region of Ssd1 (Figure 1A) (15–18). Transcripts associated with Ssd1 were enriched for mRNAs encoding cell wall biogenesis proteins and Ssd1 was shown to repress their translation (11,17,19). Ssd1 also suppresses translation of the cell cycle regulator Cln2, by binding to its transcript (20), suggesting a pathway for coordination between the cell cycle and bud growth. An emerging model from these data is that Cbk1 and Ssd1 associate with new buds as yeast cells start to divide. In this model, Ssd1 may help to ensure localized translation of cell wall remodelling proteins at the bud, suppressing translation unless Ssd1 is phosphorylated by bud-localized Cbk1 (17). Consistent with this model, Ssd1 is dispensable, whereas loss of Cbk1 is lethal when wild-type Ssd1 is present (17,21). Loss of Cbk1 results in a strong cell separation phenotype that is suppressed by deletion of *SSD1* (22–24). The inability to relieve translational repression of cell wall remodelling enzymes may prevent bud growth, leading to cell growth arrest.

How Ssd1 recognizes RNA and prevents translation is not well understood, mechanistically. Many Ssd1p-associated transcripts have a common C/U-rich sequence motif, termed the Ssd1-enriched element (SEE) (19). However, the exact binding sites of Ssd1 on these RNAs are not known. The SEE element is enriched in 5′UTRs of Ssd1-associated transcripts (19), but reporter gene experiments did not clearly identify sequence elements that confer Ssd1-dependent regulation (25). The SEE sequence element occurs internally in mRNAs, and so is unlike the 3′ terminal elements recognized by RNase II family nucleases, such as Dis3L2 that recognizes terminal oligo(U) sequences.

By combining precise *in vivo* mapping of Ssd1 RNA binding sites, structural analysis of Ssd1 and site-specific mutations *in vivo*, we provide new insights into Ssd1 function. We used UV CRAC to find transcriptome-wide Ssd1 binding sites *in vivo* at nucleotide resolution. We demonstrate that Ssd1p recognizes a bipartite element that encompasses the previously identified SEE motif, along with a second upstream motif. We present a 1.9 Å X-ray crystal structure of *S. cerevisiae* Ssd1p and show that it retains the domain architecture of RNase II proteins (1,4,26). The absence of enzymatic activity in Ssd1p is revealed to arise from both mutation of active site residues and the fixing in place of loop elements that likely regulate activity of other DIS3 family enzymes. Finally, using conservation- and structure-based mutations, we identify an RNA binding site on the outer surface of the protein. Mutations at these sites phenocopy *ssd1* deletion in stress resistance. This work gives structural, biochemical and genetic evidence for the evolution of alternative RNA recognition sites within the RNase II enzyme family.

## MATERIALS AND METHODS

### Construction of yeast strains

All strains were made in the BY4741 (S288C) background; BY4741, ssd1Δ and hsp104Δ strains were taken from the yeast gene deletion collection (27) and deletions verified by PCR. Full details of strains, oligonucleotides and plasmids are given in Supplementary File S1. All oligonucleotides and gBlocks were supplied by Integrated DNA Technologies. We referred to the *Saccharomyces* Genome Database for sequence information (28). Cloning strategies, designed using SnapGene (GSL Biotech LLC, San Diego, CA), are shared at https://doi.org/10.5281/zenodo.4191151.

To fuse the genomic copy of SSD1 with a C-terminal His–TEV–Protein A (HTP) tag, a construct was designed (SSD1–HTP–URA3selplus) containing the last 100 bp of the *S. cerevisiae* SSD1 open reading frame (ORF), an HTP tag, a *Kluyveromyces lactis* SSD1 3′UTR/terminator and URA3 selection cassette and 100 bp of the *S. cerevisiae* SSD1 3′UTR. The plasmid was synthesized by GeneArt (Thermo Fisher), cut with SfiI and integrated into the genome of BY4741 by homologous recombination after lithium acetate transformation (29). Colonies were grown on selective SC-URA plates, and two independent clones were verified by PCR.

Near-scarless integration of C-terminal HF tags (HHHHHHHHAAAADYKDDDDK), or N-terminal FH tags (DYKDDDDKAAAAHHHHHHHH), with and without deletion of the codons for the first 338 amino acids of SSD1, and site-specific mutants were made using a CRISPR/Cas9 yeast plasmid pML104 with URA selection (30). Appropriate guide RNA (gRNA) sequences were identified using the CRISPR tools available at benchling.com, and imported into CRISPR tools, courtesy of the Wyrick Lab (http://wyrickbioinfo2.smb.wsu.edu/crispr.html), to design oligonucleotides. These oligonucleotides were annealed and ligated into pML104 digested with SwaI and BclI, after growth in dam^−^*Escherichia coli*. Additional synonymous mutations within the gRNA/PAM target site were included in the repair templates for most mutant strains, as needed to prevent further cleavage after repair. Repair templates (custom gBlocks; Integrated DNA Technologies) were amplified with Phusion Polymerase (New England Biolabs) for 12 cycles using specific gBlock-amplifying primers (Supplementary File S1). BY4741 yeast were transformed and selected as described above using 500 ng of gRNA plasmid (URA3 selection) ± 250–300 ng of the relevant repair template. Clones were verified by PCR analysis and sequencing. Once confirmed, tagged strains were grown overnight on non-selective medium (YPDA) and then plated on 5-FOA agar to select for loss of the gRNA plasmid.

### Growth of yeast strains

Strains not requiring selection for auxotrophic markers (or requiring loss of a URA3 plasmid) were grown in standard YPDA or YPD (without adenine) where indicated. Selection for URA3 strains was on SC-URA agar or broth [6.9 g/l Yeast Nitrogen Base without amino acids (Formedium, CYN0405) + 0.96 g/l Synthetic Complete Dropout − URA mixture (Formedium, DSCK1009) + 2% glucose (Formedium, GLU03)]. For CRAC, cells were grown in SMM-TRP [6.9 g/l Yeast Nitrogen Base without amino acids + 740 mg/l Complete Supplement Mixture − TRP (Formedium, DCS0149) + 2% glucose]. 5-FOA plates contained 6.7 g/l Yeast Nitrogen Base without amino acids, 2% glucose, 20 mg/l each of l-uracil, l-methionine and l-histidine, 50 mg/l l-lysine and 100 mg/l l-leucine + 1 mg/ml 5-FOA (Formedium, 5-FOA01, dissolved at 100 mg/ml in DMSO) and 2% agar (Formedium, AGR05).

### Yeast phenotyping assays

We investigated the sensitivity of yeast growth, by inoculating individual colonies in 5 ml YPD broth in culture tubes with vigorous shaking at 30°C and growing overnight to late log phase.

For thermo-tolerance tests, 100 μl of each overnight culture was transferred in duplicate to separate 200-μl PCR tube strips. One strip was incubated for 30 min at 37°C and then cooled to 30°C in a thermocycler. The second strip was incubated sequentially for 30 min each at 37°C and then 50°C before cooling to 30°C in a separate block in the same machine. Serial 5-fold dilutions in water were made into a 96-well plate and dilutions were replica plated on YPD plates, grown for 2 days at 30°C. For CFW sensitivity tests, 6× 10-fold serial dilutions were made in water of late log phase cultures of each strain. Five microlitres of each dilution was pipetted onto YPDA and YPDA + 50 μM CFW plates and grown at 30°C for 2 days.

To test CFW sensitivity of the structure-based point mutants, overnight cultures grown in YPDA were harvested by centrifugation at 5000 × *g* for 1 min. Volumes used were 200 μl of the BY4741 control and volumes of mutants with equivalent cell numbers, normalized by OD_600_. Cell pellets were resuspended in 100 μl of sterile water and transferred to a UV-sterilized round-bottomed 96-well plate. Five serial 10-fold dilutions were made and a BelArt 96-well Replica plating tool was used to transfer samples onto YPDA (control) and YPDA + 50 μg/ml CFW plates (Nunc OmniTrays, Thermo Scientific, cat. no. 242811). The plates were incubated at 30°C for 2 days and scanned using an Epson Perfection V750PRO Scanner with EpsonScan v3.9.2.1 software.

### CRAC of Ssd1

In summary, two 2.86 l cultures (in SMM-TRP medium in 5-l flasks) for each of two biological replicates (independent clones) of the SSD1–HTP strains, plus one of BY4741 (untagged SSD1) as a control, were prepared from overnight pre-cultures at starting OD_600_ of ∼0.05, and shaken at 30°C until they reached an OD_600_ of 0.45. Each replicate of the SSD1–HTP strain was filtered rapidly through 0.45-μM nitrocellulose membrane filters (Millipore, HAWP09000) to collect the cells. One set of filtered cells from each biological replicate was transferred on the membranes to 5-l flasks containing 2.86 l of SMM-TRP medium pre-warmed to 42°C and shaken at 42°C for 16 min before immediate transfer of the cultures to the Megatron [UVO3 (31)] for UVC (254 nm) cross-linking for 100 s. Cells were recovered again by filtration, washed in water and transferred to 30 ml of PBS in a 50-ml Falcon tube, shaking to release the cells, removing the membranes, pelleting the cells and draining the tubes before storing at −80°C. The remaining cultures were taken straight from 30°C for cross-linking and downstream treatment as above. Extracts of the cross-linked pellets were processed into sequencing libraries as previously described (31) using 1 μl of a 1:100 dilution of 10 U/μl RNace-IT (Agilent Technologies, 400720) per sample, 22 cycles of PCR after reverse transcription and size selection of products of around 120–180 bp (average 150 bp); for full details, see protocols.io. Library concentrations were measured using the Qubit dsDNA HS Assay Kit (Q32851) and pooled at 1 nM final concentration for single-end read sequencing with an Illumina MiniSeq High Output Reagent Kit (75 cycles, FC-420-1001) on an Illumina MiniSeq System Instrument.

### CRAC data analysis

Complete code for the CRAC data analysis is available at https://doi.org/10.5281/zenodo.4191151. In brief, we adapted the single-end reads pipeline developed by the Granneman lab (32), relying on multiple tools from the pyCRAC software suite (33). Initially, the 3′ adapters were removed from the FASTQ files using flexbar and then pyBarcodeFilter.py was used to demultiplex the FASTQ files based on their barcodes. pyFastDuplicateRemover.py was used to collapse PCR duplicates based on identity of both the insert sequence and the random nucleotides in the barcodes. Collapsed FASTA files were then aligned to the yeast genome using Novoalign 2.0 (Novocraft technologies). Reads were counted using multiBamCov from bedtools (34), to transcript maps from *Saccharomyces* Genome Database (35), using the ‘abundant transcript’ data derived from (36). We added default-length 25-nt 5′UTRs and 125-nt 3′UTRs for verified ORFs whose UTRs were missing from that annotation. Bedgraph files were generated using genomeCoverageBed from bedtools (34). Pileup files, including deletions and mutations, were made using pyPileup.py running on selected Ssd1-associated transcripts. Count output gtf files were made using pyReadCounters.py, and then pyCalculateFDRs.py was used to detect enriched peaks with a false discovery rate ≤0.05. We filtered to the top 100 peaks by height and searched for enriched motifs using MEME (37). RNA-seq data from GEO (GSE148166) were similarly aligned using Novoalign 2.0 and assigned to the same transcripts with multiBamCov from bedtools (34). Statistical enrichment of transcripts in Ssd1 CRAC compared to RNA was performed using DESeq2 (38) with help from biobroom (39). Data were further analysed and visualized in R (40), using ggplot2 (41), tidyverse packages (42) and R markdown (43).

### Expression and purification

The N-terminal deletion construct of Ssd1 ΔN338 and mutants were cloned as a His-tagged fusion protein into a pET-based expression vector (Supplementary File S1). The proteins were expressed in the *E. coli* strain BL21-codon plus-RIL (*DE3*) grown in 2XTY media. Cultures were induced with 0.3 mM isopropyl β-d-1-thiogalactopyranoside overnight at 20°C. Cells were lysed using a cell disruptor (Constant Systems) in lysis buffer (20 mM Tris–HCl, pH 8.0, 200 mM NaCl, 10 mM imidazole, 1 mM β-mercaptoethanol) in the presence of protease inhibitor cocktail (Roche) and DNase I (Sigma-Aldrich). The clarified lysate was bound to Ni-NTA resin (Sigma-Aldrich) in batch or using a HisTrapHP column (Cytiva). The unbound protein was washed out using the lysis buffer and the bound protein was eluted with 20 mM Tris–HCl, pH 8.0, 200 mM NaCl, 500 mM imidazole and 1 mM β-mercaptoethanol. The protein was dialyzed in 20 mM Tris–HCl, pH 7.5, 100 mM NaCl and 1 mM dithiothreitol (DTT) in the presence of rhinovirus 3C protease to cleave off the His tag. The protein was further separated from nucleic acids using a heparin Sepharose column (Cytiva) and eluted using 20 mM Tris–HCl, pH 7.5, 1000 mM NaCl and 1 mM DTT in a salt gradient. The protein was finally purified by size exclusion chromatography (Sephadex 200 column, Cytiva) in 20 mM HEPES, pH 7.5, 150 mM NaCl and 1 mM DTT.

### RNA preparation

All RNA oligonucleotides were synthesized by Biomers GmbH and reconstituted in H_2_O to a final concentration of 1 mM (Supplementary File S1). For electrophoretic mobility shift assays (EMSAs) and fluorescence anisotropy, RNA oligomers were labelled during synthesis at the 5′ end with fluorescent dyes, DY681 and Cyanine3, respectively.

### Electrophoretic mobility shift assays

Binding reactions, containing 0.5 μM RNA 5′-labelled with fluorescent dye DY681 and increasing concentrations of Ssd1 protein, were incubated on ice in binding buffer (20 mM HEPES, pH 7.5, 150 mM potassium acetate and 5 mM magnesium acetate). After 1 h of binding, samples were mixed with native gel loading buffer containing 0.25% bromophenol blue, 0.25% xylene cyanol and 50% glycerol, and 4 μl was loaded onto an 8% native polyacrylamide gel. After 1.5 or 3 h at 2 W, at 4°C, the gel was scanned on a LICOR Odyssey fluorescent infrared scanner at 700 nm. Images were converted to greyscale using LICOR Image Studio Software.

### Fluorescence anisotropy

Fluorescence anisotropy assays were carried out in a final volume of 100 μl in black, 96-well plates using a SpectraMax M5 multimode plate reader (Molecular Devices). A total of 20 nM Cy3-labelled ssRNA (in 20 mM HEPES, pH 7.5, 150 mM NaCl, 1 mM DTT and 0.01% Tween 20) was incubated with increasing concentrations of Ssd1 ΔN338 protein for 15 and 30 min on ice. Anisotropy was measured using 530 and 565 nm wavelengths for excitation and emission, respectively. Experimentally obtained anisotropy was plotted against protein concentration to determine the equilibrium dissociation constant, *K*_D_, for binding of the labelled RNA oligomer to protein (KaleidaGraph v4.5.4 Synergy Software). The binding curves are described by the following equation and were fitted by regression analysis:

$$\begin{eqnarray*} {{r\ }} &=& {{{r}}_0}{{\ }} + \left( {{{{r}}_1} - {{{r}}_0}} \right)\nonumber\\ &&\times\frac{{({{{K}}_{\rm{d}}} + \left[ {{\rm{Rec}}} \right] + \left[ {\rm{P}} \right]) - \sqrt {{{({{{K}}_{\rm{d}}} + \left[ {{\rm{Rec}}} \right] + \left[ {\rm{P}} \right])}^2} - 4\left[ {{\rm{Rec}}} \right]\left[ {\rm{P}} \right]} }}{{2\left[ {\rm{P}} \right]}} \end{eqnarray*}$$

where *r* is the observed anisotropy, *r*_0_ is the anisotropy of free Cy3-labelled RNA, *r*_1_ is the anisotropy of fully bound RNA, [Rec] is the protein concentration, [P] is the Cy3-labelled RNA concentration and *K*_D_ is the dissociation constant for the interaction. Curve fitting for the 15 min time point used weighting based on the standard deviation. Curves are plotted as Δanisotropy, where the basal fluorescence of the probe was subtracted from all points.

### Crystallization and structure solution

Ssd1 ΔN338 was concentrated to 11.5 mg/ml and crystallized in sitting drops containing a well solution of 50 mM Tris–HCl, pH 8.0, and 25% PEG 400 at room temperature. Crystals were cryoprotected in 50 mM Tris–HCl, pH 8.0, and 30% PEG 400 and flash cooled in liquid nitrogen. Initial crystals diffracted to 3.9 Å. Crystal diffraction quality was improved after reducing the protein concentration in the sitting drops to 9.4 mg/ml. Data were collected at Diamond Light Source (DLS) on beamline I04-1. Data from crystals diffracting to 1.9 Å, with space group *P*1, were obtained and indexed and reduced using the automated data processing suite at DLS (44). The structure was solved by molecular replacement by using separate domains from Rrp44 [Protein Data Bank (PDB) ID: 2vnu (45)] and DIS3L2 [PDB ID: 4pmw (46)] with PHASER (47) in MR mode. Two molecules were found in the asymmetric unit. Sub-fragments of the structure of yeast Rrp44 (Dis3) were used as search models and RNB domains were placed first followed by the two N-terminal CSDs from Rrp44 as a single search model. The S1 domain from DIS3L2 was placed last. After initial placement of these sub-fragments, the model was refined using MORPHMODEL in PHENIX (48,49), followed by rounds of rebuilding in COOT (50) and refinement in PHENIX. The final model was assessed for quality using MOLPROBITY (51). Figures were prepared with IBS (52) and pymol (53).

### Motif conservation and evolutionary analysis

For alignment of Ssd1 binding site on SUN4 5′UTRs in *Saccharomyces*, orthologues of SUN4 in *S. cerevisiae*, *S. paradoxus*, *S. mikatae*, *S. kudriavzevii*, *S. arboricola*, *S. uvarum* and *S. eubayanus* were selected and sequences 700 nt upstream of the start codon were retrieved using orthology mapping and annotations provided by Shen *et al.* (54). These were aligned using MAFFT v7.429, option *genafpair* (55), and the sequence logo was computed with ggseqlogo (56). Ascomycete homologues were chosen from PANTHER family PTHR31316:SF0 (57), and their protein and transcript annotations were obtained from FungiDB (58). The annotation of the 5′UTR of *Candida albicans* SUN41 was adjusted to account for its 5′UTR intron (59). Motif occurrences were counted by eye and, where overlapping sequences such as CNYUCNYUCNYU were observed, these were counted as two occurrences of CNYUCNYU. For protein phylogeny, we aligned the sequences using MAFFT v7.429, option genafpair (55), computed the tree with fasttree 2.1.10 (60) and plotted the figure using ggtree (61).

To investigate the prevalence of motifs in other fungal species, we downloaded 1000 nt of genomic sequence upstream of the start codon of all genes in *C. albicans* SC5314, *Aspergillus fumigatus* Af293 and *Schizosaccharomyces pombe* 972h^−^ from FungiDB (58). We then counted the number of occurrences of CNYTCNYT in upstream sequences with Biostrings (62). We selected the list of genes with two or more CNYTCNYT occurrences in the 100-nt upstream sequence, and performed gene ontology analysis at, respectively, the *Candida* Genome Database (63), *Aspergillus* Genome Database (64) and PomBase (65).

## RESULTS

### Ssd1 associates primarily with 5′UTRs of mRNAs encoding cell wall proteins

Previous studies indicated that Ssd1 is enriched on transcripts that encode cell wall remodelling proteins (17,19), and bioinformatic analyses of these transcripts suggested a potential Ssd1 binding motif (19). To locate the precise positions of RNA binding sites of Ssd1 within its RNA targets, we applied CRAC, which requires a tandem affinity tag on the protein of interest at the endogenous locus (66). To determine whether an HTP tag at the N- or C-terminus of Ssd1 would affect its *in vivo* activity, we used two functional assays for which *ssd1* phenotypes are well characterized. CFW binds to chitin in fungal cell walls, and *ssd1Δ* strains are sensitive to CFW concentrations in the range of 10–100 μM (67). Neither N-terminal nor C-terminal tags on endogenous Ssd1 increased sensitivity of cells to CFW, whereas *ssd1Δ* and a Ssd1 truncation that lacks the first 338 residues (ΔN338 Ssd1, equivalent to the construct used for structural studies, Figure 1A) were both highly sensitive to treatment (Figure 1B). Loss of Ssd1 also reduces ‘induced thermotolerance’ in yeast, where a mild heat shock protects cells from death in subsequent severe heat shock (68). Interestingly, ΔN338 Ssd1 showed a thermal tolerance phenotype similar to wild type rather than to *ssd1Δ* (Figure 1C).

We carried out CRAC on strains with a C-terminal HTP-tagged Ssd1 followed by the 3′UTR of *K. lactis* Ssd1. Biological duplicates were grown exponentially in synthetic medium at 30°C, or following heat shock at 42°C for 16 min, a condition in which total Ssd1 binding to RNA markedly increases (69). Expression of tagged constructs was verified by western blot (Supplementary Figure S1A). Tandem affinity purification of these samples efficiently recovered cross-linked RNA (Supplementary Figure S1B), while a negative control did not. CRAC sequencing data derived from these samples were reproducible, whereas a negative control strain with no tag gave low read counts and low correlation with the Ssd1–HTP results (Supplementary Figure S1C). We compared the CRAC reads to poly(A)-enriched RNA-seq from the background (untagged) yeast strain grown in matched conditions (69), which were also reproducible (Supplementary Figure S1D).

Analysis of enrichment per mRNA by DESeq2 (38) revealed strong enrichment for a small proportion of mRNAs, with 189 mRNAs at least 2-fold enriched with 5% false discovery rate at 30°C (Figure 1D and E and Supplementary Figure S1E). Within these, 35 cell wall genes account for ∼35% of all Ssd1 reads, but only 3% of the RNA-seq reads. Ssd1-bound mRNAs that encode proteins required for cell wall biogenesis or septum remodelling include *SUN4*, *SIM1*, *UTH1*, *SCW4*, *CTS1*, *DSE2*, *CCW12* and *SRL1* (Figure 1D), in agreement with previous studies (11,17,19) (Figure 1E). These mRNAs are enriched for Ssd1 binding regardless of heat shock, despite dramatic changes in RNA expression levels and an increase in overall Ssd1 binding between conditions (69) (Figure 1D and Supplementary Figure S1E–G). In contrast, mRNAs encoding heat shock proteins are increased in their expression levels on heat shock by several orders of magnitude. However, they are not enriched in Ssd1 binding when the increase in their mRNA abundance is taken into account (Figure 1D).

We next looked at the profile of Ssd1-bound reads within individual transcripts (Figure 1F), finding that Ssd1 is overwhelmingly and reproducibly bound to 5′UTRs of its target transcripts. For the paralogous genes SUN4 and SIM1, Ssd1 reads are concentrated in exon 1, upstream of a 5′UTR intron. Some targets have a series of distinct peaks in the 5′UTR, in some cases extending into the coding sequence (CCW12, CTS1, SRL1) (Figure 1F). Additional, smaller peaks in the 3′UTR were also observed in some cases (SCW4, SRL1). These data show Ssd1 to be targeted to discrete regions of specific transcripts *in vivo*; 5′UTR binding is consistent with the reported role of Ssd1 as a repressor of translation.

### MEME analysis reveals three sequence motifs associated with Ssd1 cross-link sites

We next investigated the sequence determinants of Ssd1 binding specificity. We selected high-confidence Ssd1 binding sites by first calculating peaks in CRAC density below 5% false discovery rate by pyCRAC (33), and then further reduced background by selecting only the 100 peaks with the highest counts. Consistent with previous reports, MEME analysis of these Ssd1-associated peaks identified 59 occurrences of a general motif CNYUCNYU, similar to the previously reported SEE motif AKUCAUUCCUU (Supplementary Figure S2A) (19,25). Notably, transcripts that are highly enriched in Ssd1 binding generally have more than one CNYUCNYU motif within the 5′UTR, including most of the transcripts shown in Figure 1F. For example, UTH1 and SRL1 each have five CNYUCNYU sites in their 5′UTRs (Supplementary Figure S2B).

**Figure 2.**
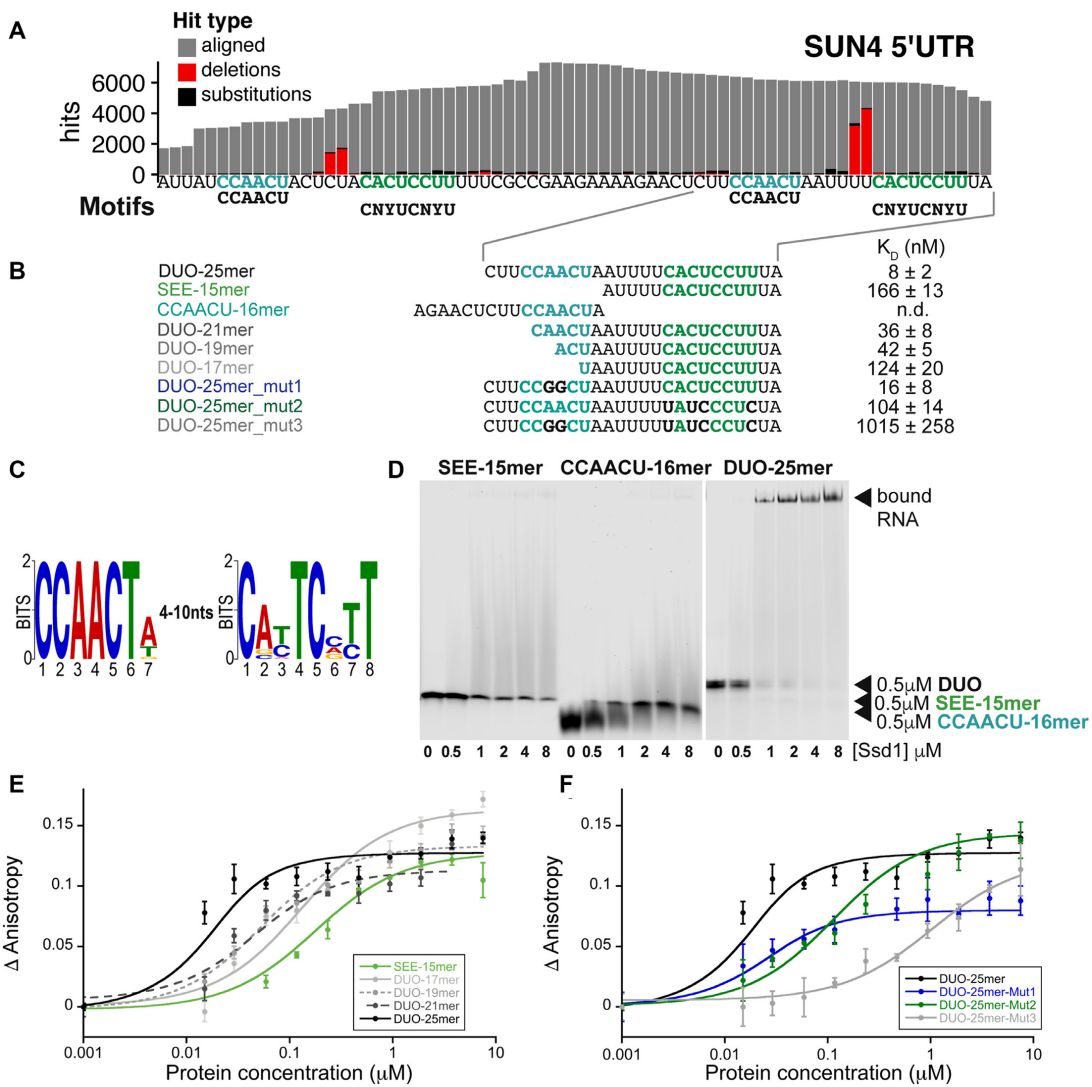
Ssd1 binds directly to a bipartite motif found in target 5′UTRs. (**A**) Zoomed-in view of CRAC read pileups on SUN4 5′UTR with CCAACU and CNYUCNYU motif positions, showing mutations (black) and deletions (red) indicative of RNA–protein cross-linking sites. (**B**) Overview of RNA oligomer sequences used in EMSA and fluorescence anisotropy binding assays with calculated *K*_D_ values in nM. (**C**) Sequence logo (in DNA alphabet) of two Ssd1-enriched motifs found by MEME analysis of the 100 top peaks in CRAC read data at 30°C. (**D**) EMSA binding assays for SUN4 5′UTR oligonucleotides. RNA probes were present at 0.5 μM. (**E**) Fluorescence anisotropy data, with fitted curves, used to calculate *K*_D_ values of different lengths of RNA derived from the SUN4 5′UTR. (**F**) Fluorescence anisotropy data, with fitted curves, used to calculate *K*_D_ values of RNA derived from the SUN4 5′UTR with mutations.

CRAC allows precise mapping of cross-linked sites because the RNA–protein cross-link leaves a moiety on the RNA after protease digestion. This ‘cross-linking scar’ can cause Superscript family reverse transcriptase to skip bases, which appear as deletions in the aligned sequences. Notably, across the dataset, a peak of higher frequency deletions 2–4 nt upstream of the CNYUCNYU motif was observed (Supplementary Figure S2B). This is exemplified by the *SUN4* 5′UTR (Figure 2A) where two nearby CNYUCNYU sites both have a high proportion of deletions a few bases upstream of the motif (Supplementary Figure S2B). This confirms that the motifs are in contact with Ssd1 *in vivo*. These deletions can be mapped only to a 4-nt region, as they are ambiguous substitutions of CUCU to CU and UUUU to UU.

We further noted that the two Ssd1-associated copies of the CNYUCNYU motif in the SUN4 5′UTR are preceded by a CCAACU motif (Figure 2A and B). Moreover, the Ssd1 cross-linking sites lie between the CCAACU motif and the CNYUCNYU motif (Supplementary Figure S2B). In contrast, a third copy of the CNYUCNYU motif in the SUN4 5′UTR does not have this upstream motif, and has far fewer Ssd1 CRAC reads (Supplementary Figure S2B). The MEME analysis of high-confidence Ssd1 binding sites found a CCAACU motif weakly enriched across the dataset, invariably appearing 4–10 nt upstream of CNYUCNYU peaks (Figure 2C and Supplementary Figure S2A). This indicates that the combination of these two motifs, as seen in *SUN4*, is a commonality among Ssd1 targets. In addition to these two motifs, a significantly enriched purine-rich motif was also observed, which we do not pursue further (Supplementary Figure S2A).

### Short sequence motifs are not sufficient for binding to Ssd1

To determine whether the CNYUCNYU motif is sufficient to bind to Ssd1, we carried out EMSAs with a synthetic RNA corresponding to one of the tandem CNYUCNYU motifs of *SUN4* 5′UTR in its native context, and recombinant ΔN338 Ssd1 (Figure 2A, B and D). However, binding of Ssd1 to this RNA oligomer was barely detectable (Figure 2D). As the MEME analysis indicated that the CCAACU motifs are also enriched in several Ssd1 targets, we tested the CCAACU motif in our EMSA but also saw no significant binding (Figure 2D). However, when we carried out the same assay using a longer ‘DUO’ RNA that encompasses both motifs, we saw strong production of a specifically shifted band (Figure 2D). This indicated that either a longer RNA or the combination of the two sequence elements, or both are required for efficient Ssd1 binding.

To better understand how these two motifs affect RNA recognition by Ssd1, we used fluorescence anisotropy to measure the binding affinity of Ssd1 to fluorescently labelled RNA ‘DUO’ oligos that encompassed both motifs, or were progressively shortened from the 5′ end to disrupt the CCAACU sequence (Figure 2B and E and Supplementary Figure S3). A 25mer oligomer encompassing both motifs bound to Ssd1 with a *K*_D_ of 8 nM, while a 15mer oligomer that encompasses only the CNYUCNYU motif had a *K*_D_ of 166 nM, consistent with the EMSA data (Figure 2D and E and Supplementary Figure S3). Intermediate-sized oligomers of 21, 19 and 17 nt showed progressively weaker binding (Figure 2B and E and Supplementary Figure S3). However, the largest changes in affinity were between the 25mer and 21mer oligomers (8 and 36 nM, respectively) and between 19mer and 17mer oligomers (42 and 124 nM, respectively) (Figure 2B and E and Supplementary Figure S3).

The loss of affinity when RNAs were shortened from the 5′ end suggested that RNA length is important. However, the sequence may also contribute to binding. To test this, we measured binding affinities for three different mutated oligos. For the first mutant, we altered the central AA bases of the CCAACU motif to GG, to maintain purines at this site but change the base. This alteration had a mild (2-fold) reduction in affinity, in a similar range observed for the DUO-19mer and DUO-17mer oligomers that have a partly truncated CCAACU motif (Figure 2B and F and Supplementary Figure S3). In the second mutation, we switched four of the conserved pyrimidine bases (C-to-U or U-to-C mutations) of the CNYUCNYU motif. This mutation substantially altered the binding affinity from the low nanomolar range to a *K*_D_ of 104 nM (Figure 2B and F and Supplementary Figure S3). The third mutation combined the alteration to the CNYUCNYU motif with that of the AA-to-GG alteration in the CCAACU motif. This mutation led to a further ∼10-fold loss of affinity. We conclude that the combined CCAACU and CNYUCNYU motif sequences are important for high-affinity binding of Ssd1. Together, these data are consistent with the CCAACU motif contributing sequence specificity in addition to the effect of RNA length.

### Ssd1 binding motifs are conserved across fungi

**Figure 3.**
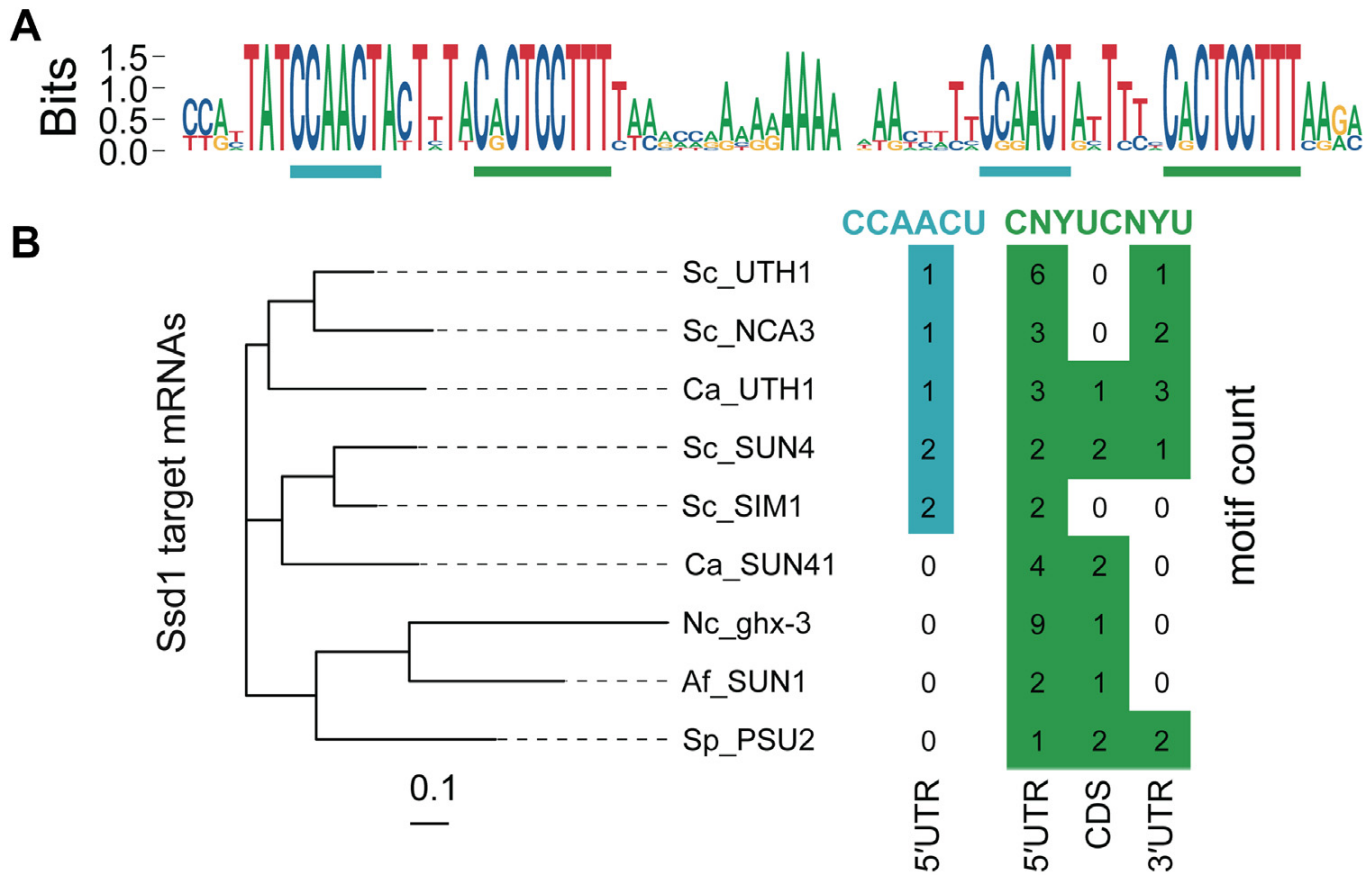
Ssd1 target sites are conserved across fungi. (**A**) Sequence logo (in DNA alphabet) of Ssd1 tandem binding site on SUN4 5′UTR as aligned from seven species of *Saccharomyces* spp. CCAACU and CNYUCNYU motifs are highlighted as in Figure 2A. (**B**) Motif counts of CCAACU and CNYUCNYU within transcripts of SUN4 homologues in select ascomycete fungi, aligned to the protein phylogenetic tree on the left. Genes are *S. cerevisiae* SUN4, SIM1, UTH1 and NCA3; *C. albicans* UTH1/SIM1 and SUN41; *Neurospora crassa* ghx-3; *A. fumigatus* SUN1; and *S. pombe* PSU2.

The Ssd1 binding site is highly conserved in homologous transcripts: we focused on the SUN4 5′UTR by aligning the upstream regions of SUN4 homologues from seven sequenced species of *Saccharomycessensu stricto* (Figure 3A). This sequence logo shows that both the CCAACU and CNYUCNYU motifs are perfectly conserved in the tandem binding site, while nearby sites are more variable. This tandem binding site was previously identified as highly conserved using a phylogenetic hidden Markov model, PhastCons (70). This confirms that specific nucleotides within these two motifs are conserved over 20 million years of evolution in the *Saccharomyces* genus.

The binding motif of Ssd1 is also conserved at longer evolutionary distances. Indeed, a sequence similar to the SEE motif was reported to be found in 5′UTRs of *S. pombe* Sts5, a homologue of Ssd1 that is also a pseudonuclease (4,71). We focused our search on homologues of SUN4; SUN4 and SIM1 are post-whole genome duplication paralogues, as are UTH1 and NCA3 (72). These putative glucanases are secreted proteins that localize to the bud scar in *S. cerevisiae* (73,74). We analysed transcript annotations of SUN4 homologues from other ascomycete fungi *C. albicans*, *N. crassa*, *A. fumigatus* and *S. pombe*. We counted the instances of CCAACU in the 5′UTR, and CNYUCNYU in the 5′UTR, CDS and 3′UTR and compared these instances with a phylogenetic tree of the proteins (Figure 3B). All of these ascomycete SUN family genes have multiple CNYUCNYU motifs in the 5′UTR or near to the start codon. For example, *S. pombe* PSU2 has one CNYUCNYU motif in the 5′UTR and two CNYUCNYU motifs in the CDS that are 14 and 38 nt downstream of the start codon. We find CCAACU motifs upstream of CNYUCNYU motifs in 5′UTRs only from *S. cerevisiae* and *C. albicans* homologues, suggesting that the CNYUCNYU part is more broadly conserved and that the bipartite RNA binding motif is particular to the budding yeast clade.

We next investigated whether CNYUCNYU motifs in 5′UTRs are associated with specific categories of genes in other ascomycetes. We selected regions of 100 nt upstream from start codons for all protein-coding genes from *C. albicans*, *A. fumigatus* and *S. pombe*, and counted the occurrences of CNYUCNYU. In each species, only a small proportion of sequences contained two or more copies of CNYUCNYU, reminiscent of Ssd1-bound transcripts in *S. cerevisiae*. Gene ontology analysis of this set of sequences revealed strong enrichment for genes encoding cell wall proteins (GO:0005618) in *C. albicans* (*P* < 10^−6^), *A. fumigatus* (*P* < 10^−4^) and *S. pombe* (*P* < 10^−3^). Other enriched gene categories include overlapping terms relating to the cell surface, cell periphery and plasma membrane. These candidate Ssd1 targets in other species include homologues of *S. cerevisiae* Ssd1-regulated genes TOS1, SCW4 and CTS1. We conclude that regulation of downstream targets by Ssd1 is conserved over >500 million years of ascomycete evolution (4,17,71).

### Ssd1 retains the domain architecture of DIS3 family nucleases

**Figure 4.**
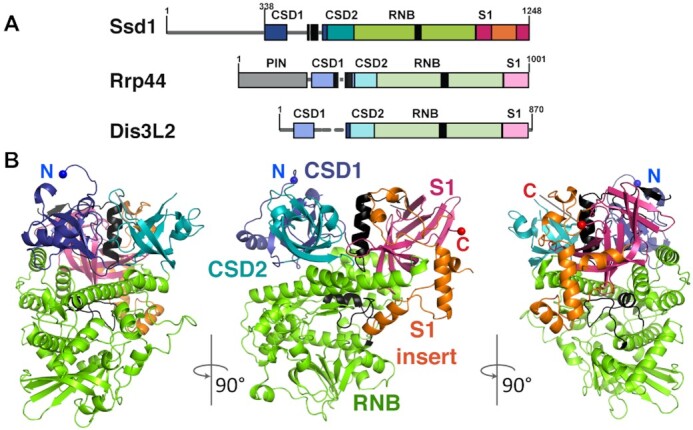
The 1.9 Å crystal structure of Ssd1 reveals conservation of fold with RNase II enzymes. (**A**) Comparison of domain structures of Ssd1 and the related proteins Rrp44 (Dis3, numbering is for yeast) and Dis3L2 (numbering is for mouse). Boxes indicate folded domains with separating grey lines indicating natively unstructured regions. Black boxes equate to features coloured black in the structural figures. (**B**) Structure of Ssd1 observed from three different viewpoints: N-terminal side view, front view and C-terminal side view. The domains are coloured to match those in (A).

The interaction data showed that Ssd1 binds to specific, highly conserved RNA sequences internal to fungal 5′UTRs. However, Ssd1 is closely related to Dis3L2 and more distantly to Rrp44 (Figure 4A), enzymes that bind and cleave RNA 3′ termini within catalytic sites that are molecular cul-de-sacs. We therefore hypothesized that the RNA binding mechanism of Ssd1 to internal sites is distinct from the 3′ terminal binding of related, but active, RNase II family members.

To better understand the molecular basis for the function of Ssd1 as an RBP, we solved the crystal structure of the core folded domains of Ssd1. This truncated protein lacks the first 338 residues that are predicted to be natively unstructured (Figure 4A) but is sufficient to bind Ssd1-specific RNA motifs *in vitro* (Figure 2). Despite the low sequence identity between Ssd1 and yeast Rrp44 or mouse Dis3L2 (22% and 27%, respectively), the structure was solved to 1.9 Å by molecular replacement using fragments of both Rrp44 and Dis3L2.

The overall structure of Ssd1 retains the RNB family domain organization: two N-terminal β-barrel cold shock domains (CSD1 and CSD2) sit at the mouth of a funnel-shaped RNB fold. Opposing CSD1 and CSD2 is a C-terminal β-barrel S1 domain (Figure 4A and B). Several loop regions (415–484, 492–497, 530–535, 562–578, 1190–1193) could not be assigned in the structure. In total, around 12% of the structure was not visible in the map and could not be built (Supplementary Figure S4). The refined model shows good stereochemistry with final *R*_work_ and *R*_free_ of 20.5% and 22.4%, respectively (Table 1).

**Table 1.** Statistics for X-ray crystallographic data reduction and refinement

	Native
**Data collection**	
Beamline	I04-1
Wavelength (Å)	0.91587
Space group	*P*1
Unit cell	*a* = 69.5 Å, *b* = 74.0 Å, *c* = 106.5 Å
	*α* = 91.3°, *β* = 91.8°,*γ* = 117.6°
Resolution	61.5–1.9 (1.94–1.9) Å
Reflections	388 684 (19 110)
Unique reflections	144 175 (7081)
*R*_meas_ (%)	7.6 (138.7)
CC (1/2)	0.998 (0.552)
Completeness (%)	98.0 (97.3)
Mean *I*/*σ*_*I*_	7.3 (0.8)
Multiplicity	2.7
**Refinement**	
*R*_work_/*R*_free_	20.5%/22.4%
RMS bonds	0.004
RMS angles	0.656
Ramachandran	
Allowed	98%
Partially allowed	2%
Disallowed	0
Total number of atoms	13 015
Protein atoms	12 049
Water/ligands	606

Relative to bacterial RNase II enzymes, Ssd1 contains two insertion elements that are likely to be functionally important. CSD1 is interrupted by an insertion in the loop between strands β4 and β5 (Figure 4 and Supplementary Figure S4), only a portion of which could be assigned in the model (Supplementary Figure S5A). A similar insertion is present at the same position in both Rrp44 and Dis3L2, so this may be a feature of DIS3 family proteins (Supplementary Figure S4). An additional, Ssd1-specific insertion (residues 1119–1204) interrupts the S1 domain (Figure 4 and Supplementary Figure S4).

### Structural and sequence changes underlie loss of nuclease activity

**Figure 5.**
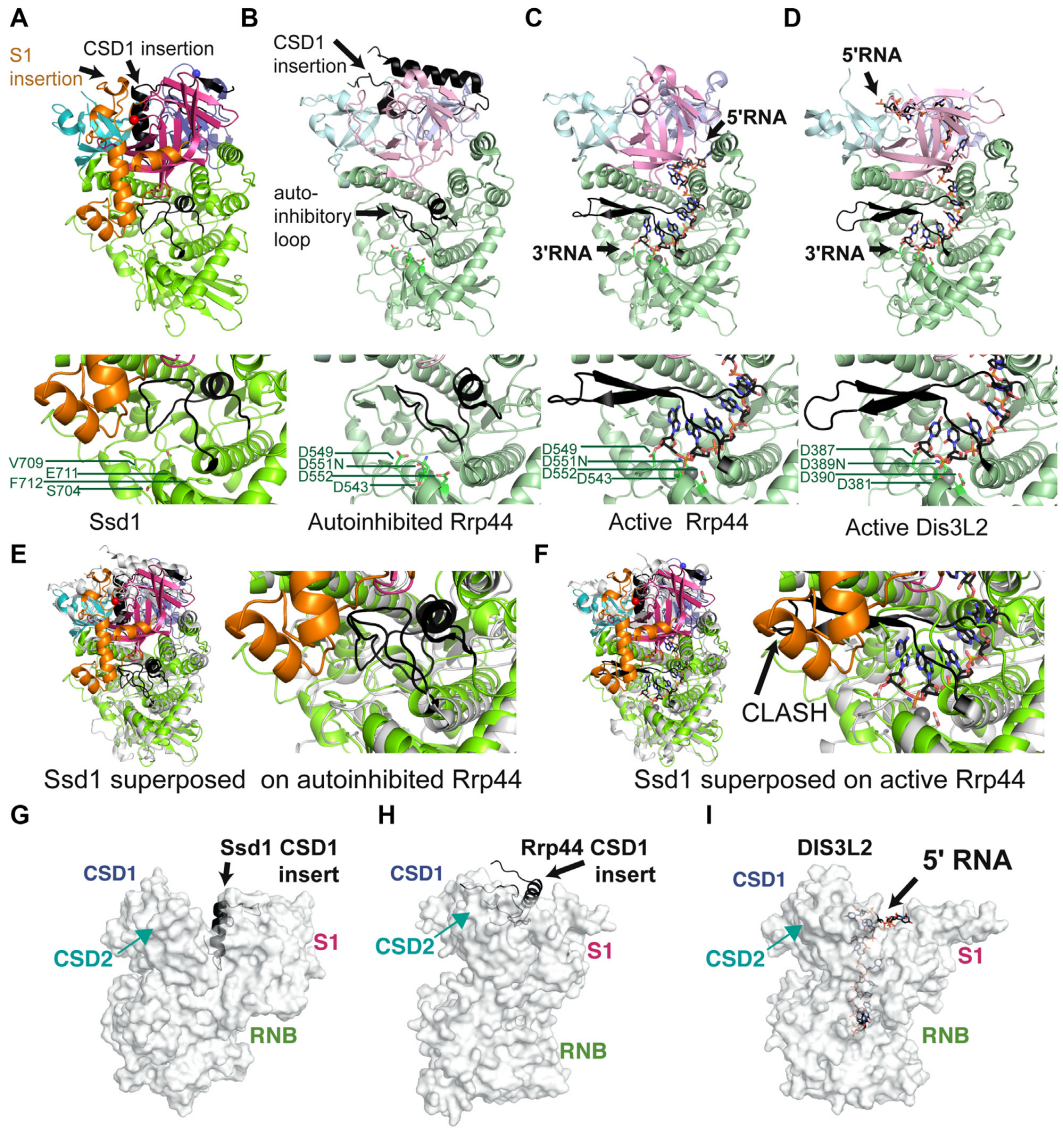
Ssd1-specific structural alterations lock it in an inactive state. Comparison of Ssd1 (**A**) with autoinhibited Rrp44 (**B**) (2wp8), active Rrp44 (**C**) (2vnu) and active Dis3L2 (**D**) (4pmw) using the C-terminal side view. Domains are coloured to match Figure 4A. The CSD1 insertion and autoinhibitory loop are shown in black. RNA bound to active Rrp44 and Dis3L2 is shown as sticks, with black carbon atoms and with Mg^2+^ ions shown as grey spheres. Under each structure is a zoomed-in view of the equivalent active site residues, shown as sticks. The PIN domain of Rrp44 and associated exosome subunits are omitted for clarity. (**E**) A zoomed view of the active site region of autoinhibited Rrp44 superposed on Ssd1. (**F**) A zoomed view of the active site region of RNA-bound Rrp44 superposed on Ssd1, showing clashes between the reordered autoinhibitory element (black) and the Ssd1-specific S1 insertion (orange). The equivalent segment in Ssd1 is coloured green and occupies the same space as the 3′ end of the RNA. (**G**) Insertion of the CSD1 insert (black) into the funnel region between the CSD and S1 domains of Ssd1 (grey surface). The view is the ‘front’ view from Figure 4B. (**H**) Insertion of the CSD1 insert (black) into the funnel region of autoinhibited Rrp44 (grey surface). (**I**) Similar view of Dis3L2 compared to Ssd1 and Rrp44 in (G) and (H), showing the path of the RNA through the funnel.

Four structural alterations contribute to the loss of nuclease activity in Ssd1. First, active RNase II nucleases have a cluster of four acidic residues that coordinate a divalent cation required for catalysis (26,75). These are absent in Ssd1 (5), with the structurally equivalent residues being Ser704, Val709, Glu711 and Phe712; this configuration is unable to coordinate a divalent cation (Figure 5A).

Of the available structures of eukaryotic RNase II nucleases, Ssd1 most closely resembles the conformation of Rrp44 when in a complex with Rrp41 and Rrp45 of the exosome core complex (compare Figure 5A and B) (76). In this structure, a loop segment within the RNB domain of Rrp44 forms an α-helix that blocks the channel normally occupied by the RNA substrate during catalysis (Figure 5B). A similar configuration is observed in Ssd1, representing a second structural change that contributes to loss of activity (Figure 5A and Supplementary Figure S5B). This segment blocks the lower tract of the residual active site. In contrast, in structures of Rrp44 and Dis3L2 engaged with RNA substrates, this loop is rearranged to form a β-hairpin motif outside of the active site, allowing RNA substrates to be accommodated (Figure 5C and D) (45,46). Indeed, this active conformation is also observed in other RNase II nucleases such as Dss1 and *E. coli* RNase II (Supplementary Figure S5C and D) (26,77).

In Rrp44, the mobile autoinhibitory element can switch between active and inactive states (Figure 5B and C). In contrast, this segment in Ssd1 is fixed in place by a third structural change: the insertion segment in the S1 domain that is apparently unique to Ssd1 (Figure 5A and E, orange segment). The S1 insertion packs against the RNB domain and stabilizes the inhibitory conformation of the loop (Figure 5E). Superposition of Ssd1 on active Rrp44 shows that the S1 insertion would have a steric clash with the autoinhibitory segment in the active configuration (Figure 5F). We conclude that the S1 insertion locks the autoinhibitory segment in place, ensuring a fixed, inactive conformation.

A fourth structural element prevents access of RNA to the former active site of Ssd1. Active enzymes, such as Dis3L2 (Figure 5D), bacterial RNase II (Supplementary Figure S5D) and human DIS3 (Supplementary Figure S5E), anchor substrate RNA at the mouth of the funnel created by CSD1, CSD2 and S1 domains, allowing the RNA to thread into the active site (26,46,78). The CSD1 insertion in Ssd1, which is only partially assigned in this structure, folds into an α-helix that blocks the mouth of the funnel, excluding RNA binding at this surface (Figure 5G). Once again, the S1 insertion element stabilizes this conformation by packing against the CSD1 insertion (Figures 4B and 5A). A CSD1 insertion at an equivalent position is not present in bacterial RNase II but is present in Dis3L2, human DIS3 and yeast Rrp44. In Dis3L2, the insertion was engineered out of the construct used for crystallization and so cannot be observed (46). A large portion of the Rrp44 CSD1 insertion is ordered in the autoinhibited structure of yeast Rrp44 and, similar to Ssd1, blocks the upper cavity (76) (Figure 5H). In Dis3L2 and other RNase II enzymes, this cavity is required for RNA access (Figure 5I and Supplementary Figure S5C and D). It should be noted, however, that RNA substrates typically access yeast Rrp44 active site by tunnelling between CSD1 and the RNB domain (Figure 5C), while the human homologue DIS3 has been observed to bind RNA in a similar mode to Dis3L2 (Supplementary Figure S5E) (78,79). The structural elements that block RNA access to the central channel of Ssd1 may have evolved from regulatory switches in ancestral proteins that have become fixed in the ‘off’ state.

### The cold shock domains of Ssd1 have a highly conserved surface

As the insertion elements in Ssd1 block the residual active site of the RNase II fold, Ssd1 cannot bind RNA using the same mode as its enzyme relatives. We examined the surface properties of the protein to search for alternative RNA binding sites. Our previous evolutionary analysis of fungal Ssd1 and Dis3L2 homologues places Ssd1 as the sole *S. cerevisiae* homologue of Dis3L2, and shows that the CSDs are highly conserved in Ssd1 homologues in ascomycete and basidiomycete fungi (4). Based on this analysis, we segregated high-confidence Ssd1 sequences from other Dis3L2 homologues that retain negatively charged residues at the active site; i.e. we excluded sequences that are likely to be active nucleases. Using a multiple sequence alignment consisting of 91 high-confidence Ssd1 homologues across fungi (Supplementary File S2), surface conservation was calculated using the CONSURF server (80,81). This revealed an extensive, conserved surface around the two CSD domains and the RNB domain (Supplementary Figure S6A). The conserved area coincides with a large surface patch of positive charge, consistent with binding to nucleic acids (Supplementary Figure S6B). This candidate RNA binding surface provides further evidence that RNA binding by Ssd1 is distinct from substrate recognition by Rrp44 and Dis3L2 enzymes.

### Ssd1 likely uses an outer surface for RNA binding

**Figure 6.**
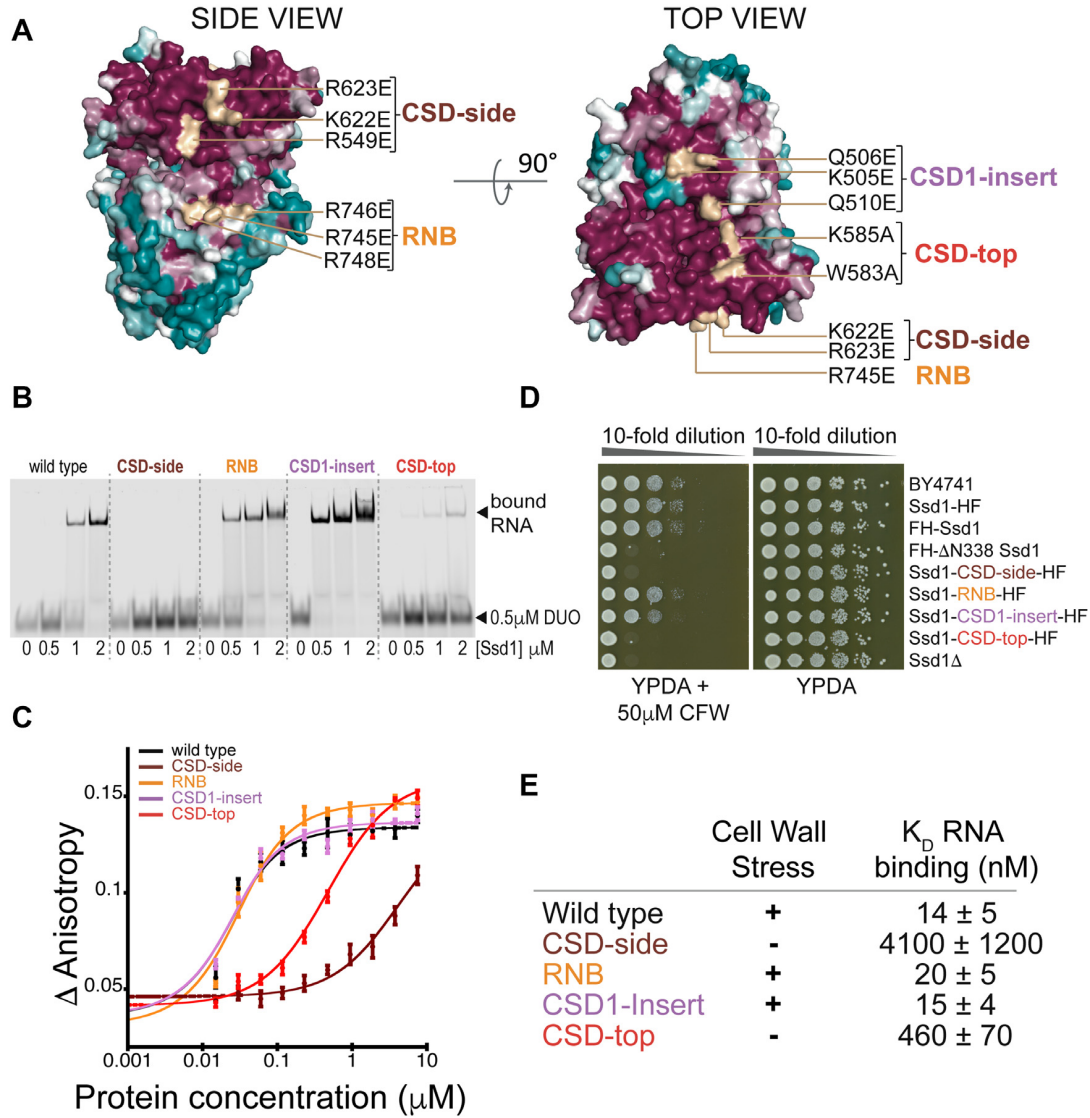
The RNA binding site is likely to be located on the cold shock domains. (**A**) Overview of clusters of mutations that are candidates for RNA binding sites. Two surface views of Ssd1 are shown coloured by conservation over a wide range of fungal species. Surface patches matching the clusters are coloured wheat. A ‘top’ view of the protein is shown via a 90° rotation. Purified recombinant mutant proteins were tested for RNA binding by EMSA (**B**) and fluorescence anisotropy (**C**). (**D**) Phenotyping assay of the equivalent mutants in yeast, using CFW as a cell wall stress. (**E**) Comparison of *in vivo* phenotype with RNA binding constants calculated from fitted curves in (C).

Based on the conserved surfaces of Ssd1, we designed four clusters of mutations to determine whether these regions contribute to RNA binding. We purified mutant proteins with two or three mutated residues on the residual RNB domain (RNB), two surfaces of the CSDs (CSD-side, CSD-top) and a patch arising from the CSD1 insertion sequence (CSD1-insert) (Figure 6A and Supplementary Figure S7A). All four mutants showed identical *in vitro* properties to the native protein on size exclusion chromatography and no or mild alterations to their thermal denaturation profiles, indicating that they are correctly folded (Supplementary Figure S7A–C). Mutation of either the CSD-side or CSD-top clusters substantially reduced RNA binding, as assessed by EMSA and fluorescence anisotropy (Figure 6B and C and Supplementary Figure S7D). In contrast, little effect was seen for the RNB and CSD1-insert clusters.

We used CRISPR–Cas9 to introduce the same set of mutations into the chromosomal *SSD1* locus, using the strain expressing Ssd1-HF. Cells expressing only Ssd1-CSD-side-HF or Ssd1-CSD-top-HF showed reduced viability when exposed to cell wall stress, similar to the *ssd1*Δ phenotype (Figure 6D and E). In contrast, strains expressing mutant proteins that retain RNA binding activity (Ssd1-CSD-RNB-HF and Ssd1-CSD1-insert-HF) resisted stress as well as the wild type (Figure 6D and E). All mutant proteins had similar expression levels to otherwise wild-type, tagged Ssd1, indicating that they are stable *in vivo* (Supplementary Figure S7E).

We conclude that the RNA binding site of Ssd1 is located on the highly conserved outer surface of the cold shock domains and is required for Ssd1 function *in vivo*.

## DISCUSSION

Previous studies indicated a role of Ssd1 in translational regulation of cell wall proteins and localization of proteins encoded by associated transcripts, with stress sensitivity due to defective cell walls and/or failure to repair bud scars in ssd1 deletion or truncation strains (11,15,17). Supporting these observations, our data show that over a third of Ssd1-associated CRAC reads were associated with transcripts encoding cell wall proteins, primarily in 5′UTRs. The tight (low nM) binding to specific sequences in 5′UTRs may physically block scanning of translation pre-initiation complexes. Regardless of the mechanism, translational repression is likely to be conserved among fungi that encode Ssd1 homologues. Ssd1 may also coordinate the cell cycle and bud growth, with effects on ploidy, through low-abundance regulatory targets such as Cln2 in *S. cerevisiae* (20) or Nrg1 in *C. albicans* (82). However, these very low abundance mRNAs were not well detected in CRAC.

**Figure 7.**
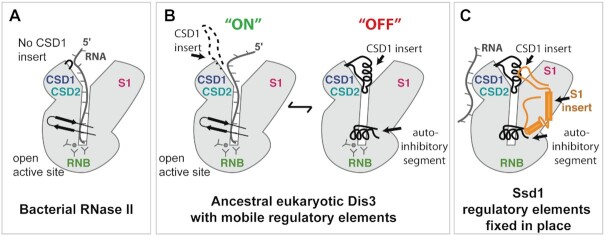
Evolution of RNase II enzymes and pseudoenzymes. (**A**) Bacterial RNase II has a domain structure that is conserved in evolution but lacks the eukaryote-specific insertions. RNA accesses the active site by funnelling into the core of the protein. (**B**) An ancestral Dis3/Dis3L2 enzyme may have acquired mobile regulatory elements that allow the enzyme to be finely regulated. The ‘ON’ state resembles that of the bacterial enzyme, while the ‘OFF’ state uses the CSD1 insertion and the autoinhibitory segment to block the funnel. (**C**) The autoinhibitory elements have been fixed in place in Ssd1 by the S1 insertion element and the active site residues have been lost. A new RNA binding site has been acquired.

The Ssd1 structure reveals an inactive pseudonuclease, in which the ancestral path of RNA into the funnel of the active site is blocked by fixed structural elements. These fixed elements are informative about the evolutionary history of this family of proteins. Bacterial RNase II proteins do not appear to have regulatory elements such as an insertion in CSD1 (Figure 7A). Moreover, the autoinhibitory loop is in an open conformation (Supplementary Figure S5D), as observed in many structures of related nucleases, particularly when RNA substrates are present (Figure 5C and D and Supplementary Figure S5C and E). In contrast, the closed, autoinhibited conformation was previously observed only for Rrp44 (Figure 5B) (76). The combination of the autoinhibitory segment and the CSD1 insertion, which is able to block the top of the funnel (at least in Ssd1 and Rrp44; Figure 5G and H), suggests that ancestral Dis3 family enzymes may have acquired these two mobile elements to facilitate regulation, by switching between a closed, autoinhibited form and an open, active form (Figure 7B). In Ssd1 homologues, these segments have been trapped in the ‘off’ state by the Ssd1-specific S1 insertion that packs against both the CSD1 insertion and the autoinhibitory loop (Figure 7C). It is unlikely that the CSD1 insertion and autoinhibitory element of Ssd1 are dynamic as they show low *B* factors that are comparable to the rest of the core of the protein (Supplementary Figure S8A). Similarly, we think it is unlikely that this ‘off’ conformation arises from crystal contacts. The S1 insertion that holds both elements in place does contribute a crystal contact. However, the S1 insertion buries 2364 Å^2^ of the RNB and S1 domain surfaces, a substantial surface area with extensive hydrophobic interactions. This argues against the S1 insertion being a dynamic element (Supplementary Figure S8B and C).

An important consequence of Ssd1 having acquired a permanent ‘off’ state is that the funnel-shaped RNA binding site that recognizes RNA 3′ ends is blocked. Instead, Ssd1 has gained a new RNA binding site that allows it to bind to sequences internal to transcripts. It is likely that the new RNA binding site is a conserved, positively charged region on the outer face of the two CSDs (Figures 6 and 7C and Supplementary Figure S6). Our previous evolutionary analysis indicated that Ssd1 is the closest yeast homologue of Dis3L2 and that loss of nuclease function in Dis3L2 homologues has occurred independently in multiple fungal lineages (4). These analyses also indicated that the CSDs are the most highly conserved part of Ssd1 in most fungi. We speculate that a Ssd1 ancestor was a bifunctional RNA degrading nuclease and RBP, and that the latter function has been preserved in preference to the nuclease activity. This acquired RNA binding activity is required for the *in vivo* function of Ssd1, presumably through translational repression. Given that Ssd1 is a virulence factor for many fungal pathogens, understanding both the molecular mechanism and the cellular functions of this translational block is an important goal for future work.

## DATA AVAILABILITY

The complete pipeline and intermediate data for CRAC data analysis, including figure construction, are available at https://doi.org/10.5281/zenodo.4191151 along with cloning strategies. A detailed protocol for the CRAC experiment is given at https://dx.doi.org/10.17504/protocols.io.5ppg5mn.

Coordinates for the Ssd1 structure were deposited in the PDB (PDB ID: 7AM1). CRAC datasets have been deposited on GEO, accession number GSE159835.

## Supplementary Material

gkab615_Supplemental_Files
