# Supplementary material for: Yeast Ssd1 is a non-enzymatic member of the RNase II family with an alternative RNA recognition site
Source: Nucleic Acids Res. 2021 Jul 24;50(5):2923–37. doi: 10.1093/nar/gkab615 (PMC8934651; doi:10.1093/nar/gkab615)
Supplement: gkab615_Supplemental_Files [file gkab615_supplemental_files.zip › NARresub-Supplementary figures_and_methods_AC07062021.pdf]

Supplementary figures and methods for:

Yeast Ssd1 is a non-enzymatic member of the RNase II family with an alternative RNA recognition interface

Rosemary A. Bayne, Uma Jayachandran, Aleksandra Kasprowicz, Stefan Bresson, David Tollervey, Edward W. J. Wallace and Atlanta G. Cook

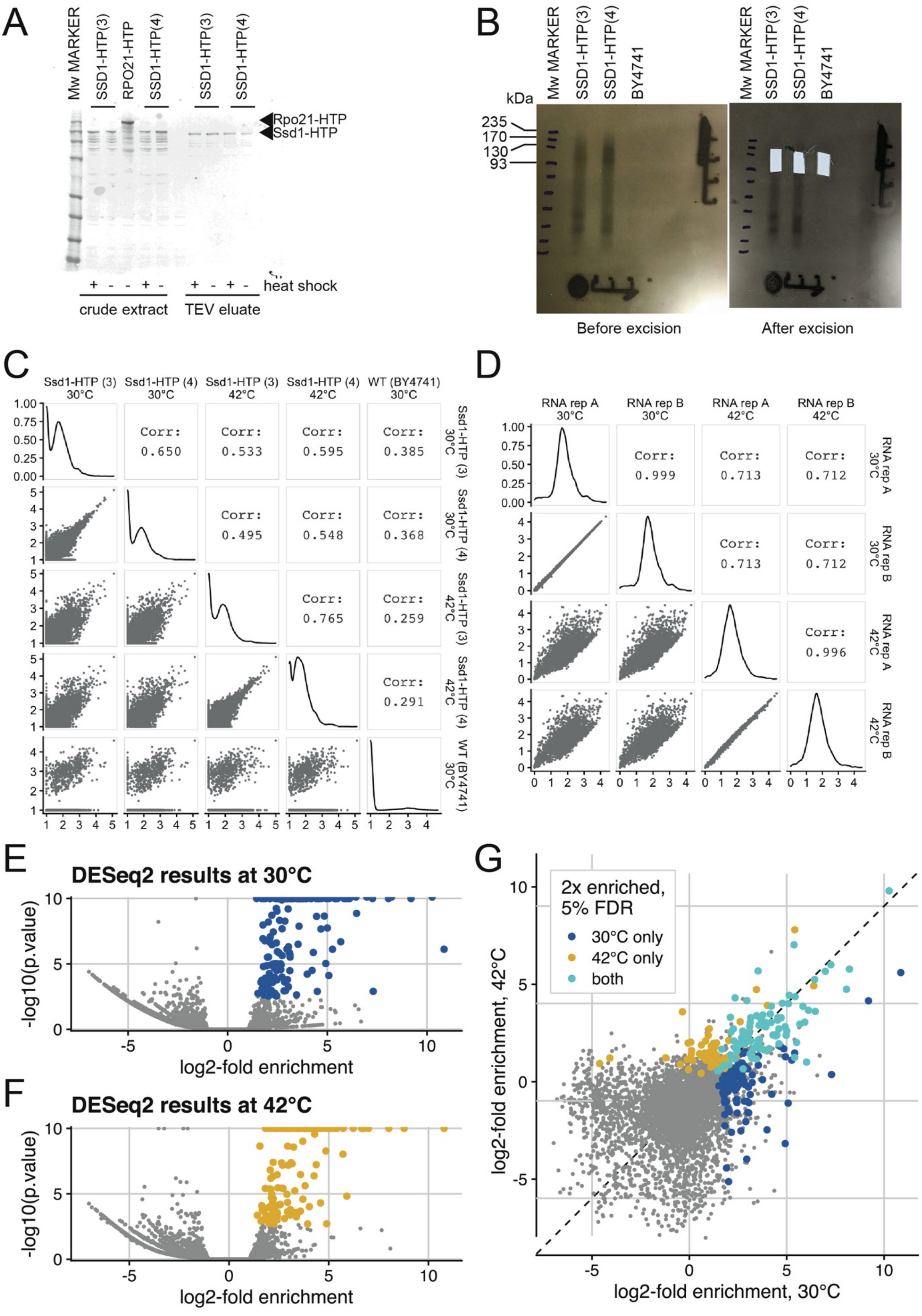

A
Mw MARKER
SSD1-HTP(3)
RPO21-HTP
SSD1-HTP(4)
SSD1-HTP(3)
SSD1-HTP(4)
Rpo21-HTP
Ssd1-HTP
+ - - + - + - + -
heat shock
crude extract
TEV eluate
B
kDa
235
170
130
93
Mw MARKER
SSD1-HTP(3)
SSD1-HTP(4)
BY4741
Before excision
After excision
C
Ssd1-HTP (3) 30°C
Ssd1-HTP (4) 30°C
Ssd1-HTP (3) 42°C
Ssd1-HTP (4) 42°C
WT (BY4741) 30°C
Corr: 0.650
Corr: 0.533
Corr: 0.595
Corr: 0.385
Corr: 0.495
Corr: 0.548
Corr: 0.368
Corr: 0.765
Corr: 0.259
Corr: 0.291
D
RNA rep A 30°C
RNA rep B 30°C
RNA rep A 42°C
RNA rep B 42°C
Corr: 0.999
Corr: 0.713
Corr: 0.712
Corr: 0.713
Corr: 0.712
Corr: 0.996
E
DESeq2 results at 30°C
-log10(p.value)
log2-fold enrichment
F
DESeq2 results at 42°C
-log10(p.value)
log2-fold enrichment
G
2x enriched, 5% FDR
30°C only
42°C only
both
log2-fold enrichment, 42°C
log2-fold enrichment, 30°C

Figure S1. (**A**) Western blot of the crude extracts and TEV eluted CRAC samples was probed with rabbit anti-TAP antibody (ThermoFisher CAB1001; 1/5000 dilution) followed by donkey anti-rabbit Dylight680 antibody (ThermoFisher SA5-10042; 1/10000 dilution) and imaged on a LICOR Odyssey CLx Infrared Imaging System. (**B**) Autoradiograph of ^{32}P - labelled SSD1-RNA complexes before and after extraction of the smear above the Ssd1 protein used to prepare the libraries. (**C**) Pairwise comparison of read densities on verified coding transcripts from CRAC datasets, in units of log10 of transcripts per million (TPM). (**D**) Pairwise comparison of read densities on verified coding transcripts from RNA-seq datasets, also in log10(TPM). (**E**) Ssd1 enrichment, results of DESeq2 analysis comparing Ssd1-bound RNA (CRAC) with poly(A)-enriched RNA-seq. Volcano plot comparing log2-fold enrichment with raw p-value for every mRNA measured at 30°C. Genes with over 2x enrichment at 5% FDR are highlighted; p values below 10^-10 are compressed to display at the top of the plot. (**F**) Similar volcano plot at 42°C. (**G**) Comparison of Ssd1 log2-fold enrichment at 30°C and 42°C.

A

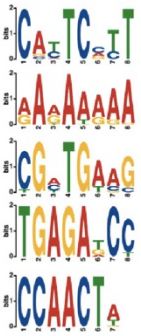

Meme	LOGO	E value	No. of nucleotides	No. of sites
1		2.4e-037	8	59
2		9.8e-010	8	59
3		2.9e-004	8	15
4		1.5e-001	8	9
5		7.5e-001	7	16

B

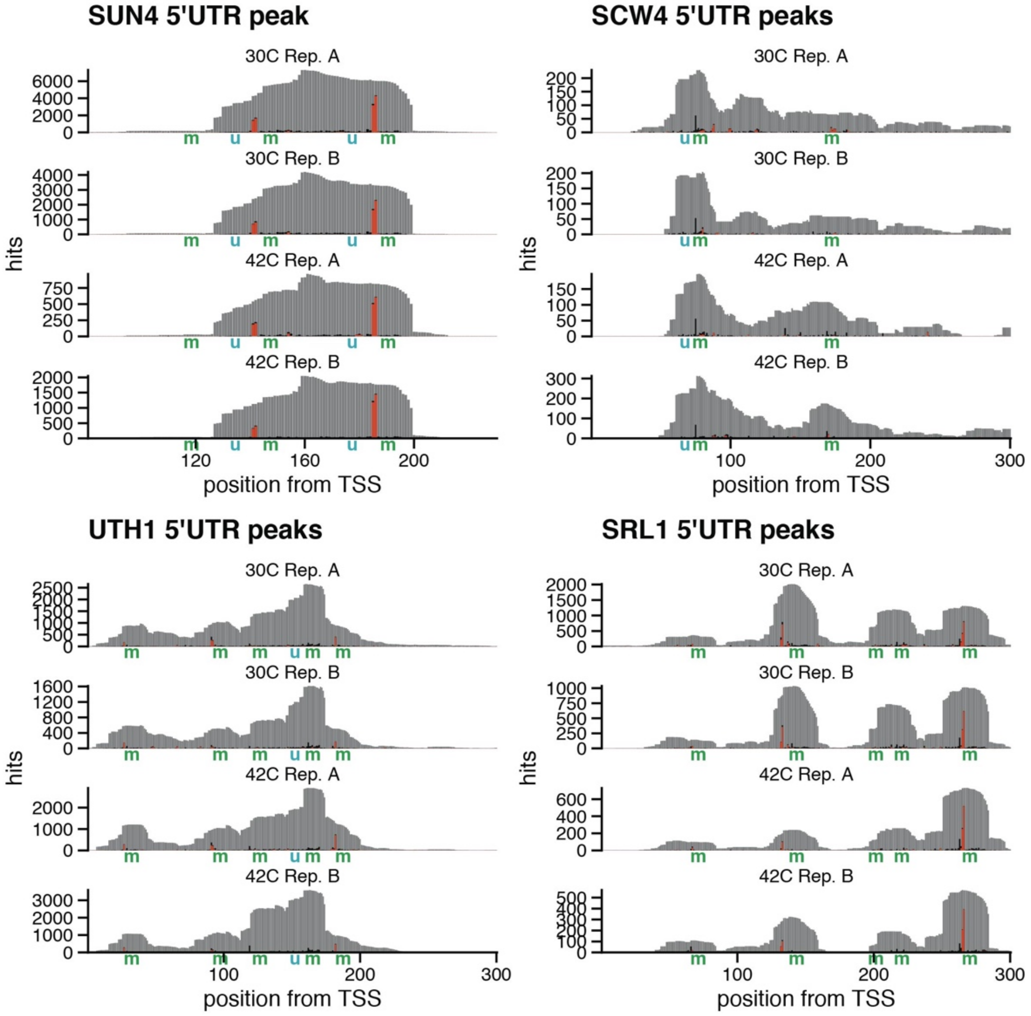

Figure S2. (**A**) Logos of sequence motifs enriched in transcripts around CRAC crosslink sites. The top five logos from MEME analysis of 100 highest-count peaks at 30°C are shown. (**B**) Pileup plots of CRAC hits which match the genome (grey) or have mutations (black) or insertions (red), showing all 4 replicates on 5′ ends of four selected transcripts, SUN4, SCW4, UTH1, and SRL1. CNYUCNYU positions are marked with "m" for motif, and CCAACU positions with "u" for upstream.

15 min incubation

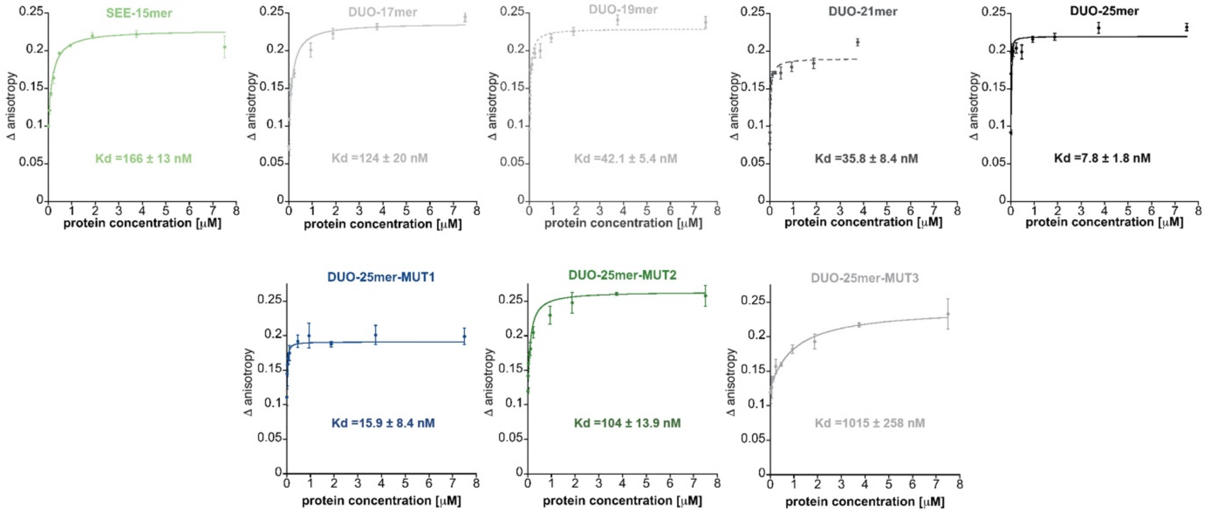

30 min incubation

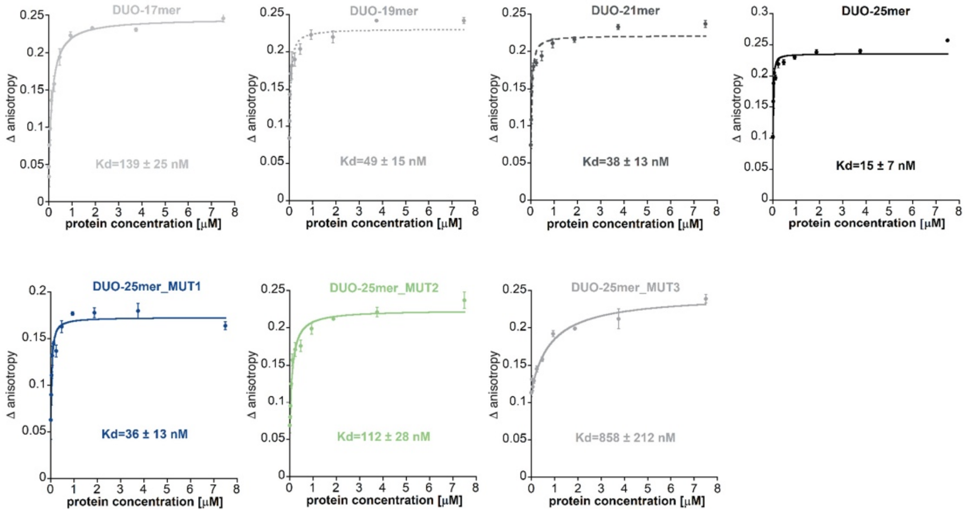

Figure S3. Fluorescence anisotropy data used in Figure 2 shown as individual experiments with fitted curves. Data from an additional set of experiments carried out with longer incubation times are included for comparison. Graph fitting for measurements at 15 mins were weighted by standard deviation.

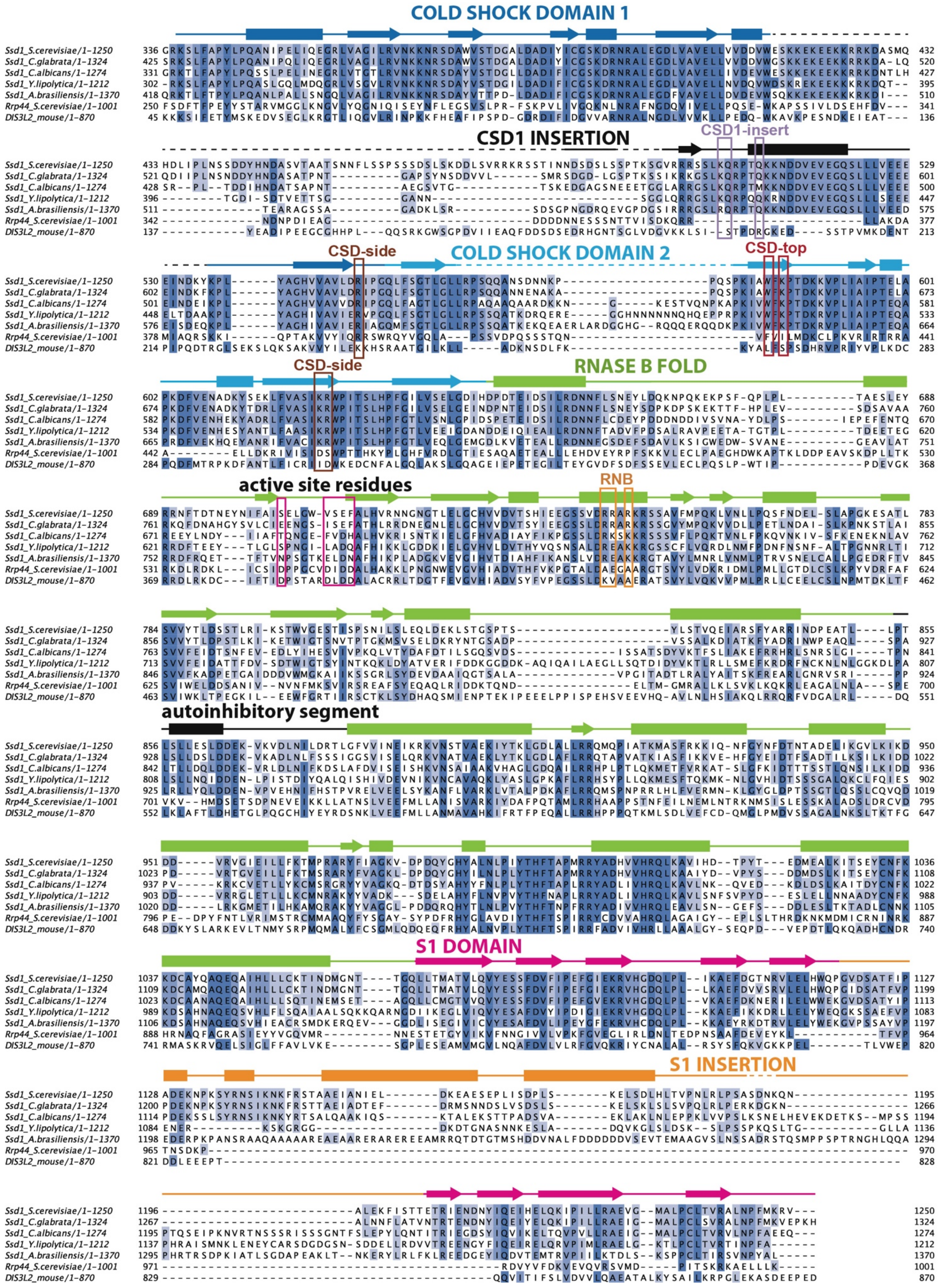
COLD SHOCK DOMAIN 1
CSD1 INSERTION
CSD1-insert
CSD-side
COLD SHOCK DOMAIN 2
CSD-top
RNASE B FOLD
active site residues
RNB
autoinhibitory segment
S1 DOMAIN
S1 INSERTION
Ssd1_S.cerevisiae/1-1250
Ssd1_C.glabrata/1-1324
Ssd1_C.albicans/1-1274
Ssd1_Y.lipolytica/1-1212
Ssd1_A.brasiliensis/1-1370
Rrp44_S.cerevisiae/1-1001
DIS3L2_mouse/1-870

Figure S4. A multiple sequence alignment that includes several Ssd1 homologues from Fungi as well as yeast Rrp44 and mouse Dis3L2. The alignment is coloured based on percent identity. Secondary structure elements for alpha helices (rectangles) and beta strands (arrows) are shown above the sequence, with unassigned segments represented by dotted lines. Pink boxes indicate residues equivalent to active site signature in active RNB family enzymes. Purple, brown, red, and orange boxes indicate the clusters of mutants used to map the RNA binding sites.

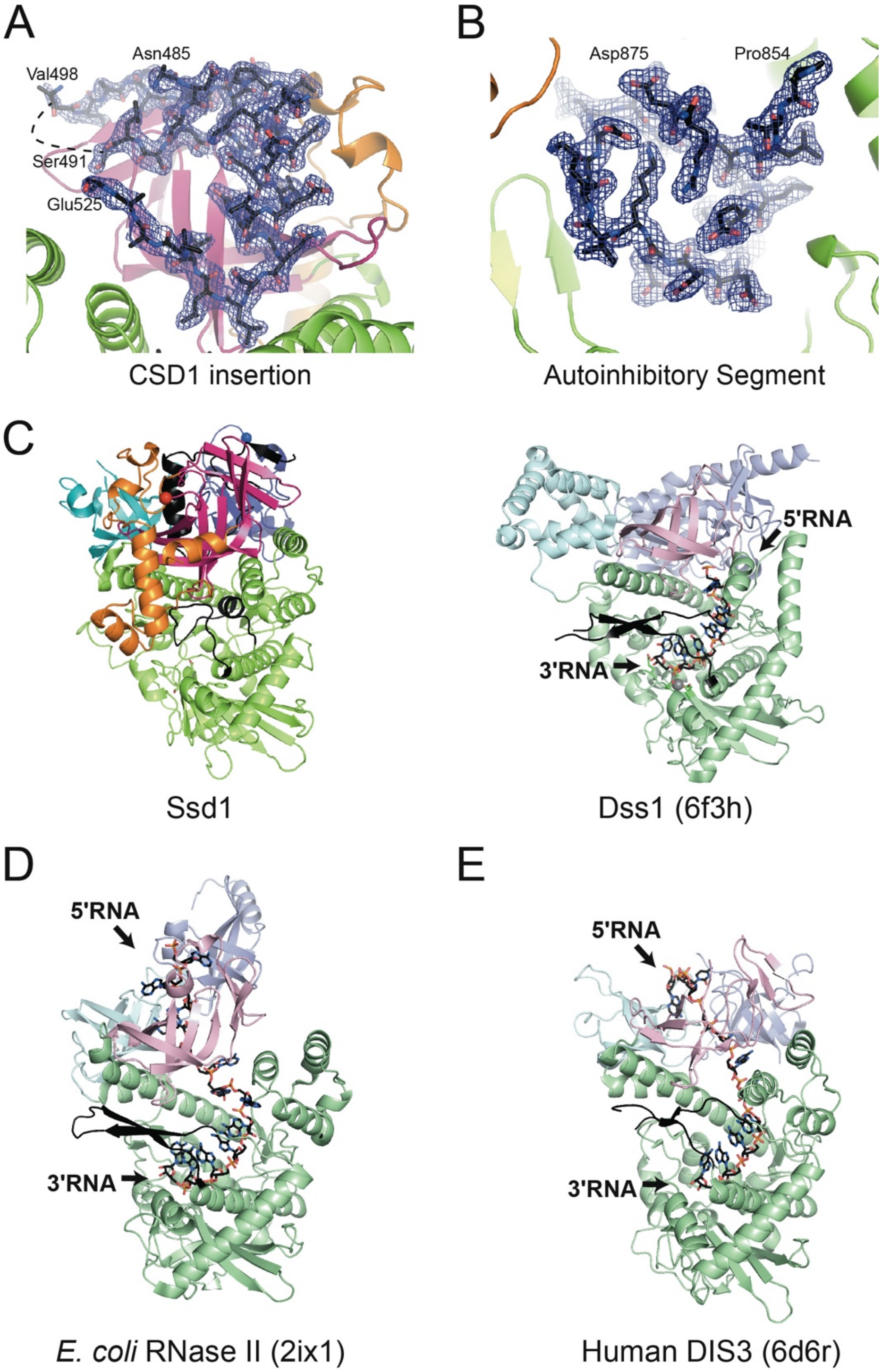

Figure S5. (**A**) Electron density around the CSD1 insertion. The view is the same as the left view in Figure 1B but with the two CSD domains removed for clarity. (**B**) Electron density around the autoinhibitory segment. The view is the same as the right-hand view in Figure 1B but rotated through 90° around the x-axis, for clarity. (**C**) Ssd1 is compared by superposition with RNA-bound Dss1 (PDBID 6f3h). (**D**) *E. coli* RNase II (PDBID 2ix1) bound to RNA is shown in the same orientation of Ssd1 as in (**C**). (**E**) Human DIS3 (PDBID 6d6r) from the exosome structure containing Mtr4 and RNA is shown in the same orientation as (**C**). The exosome subunits and part of the RNA are removed for clarity.

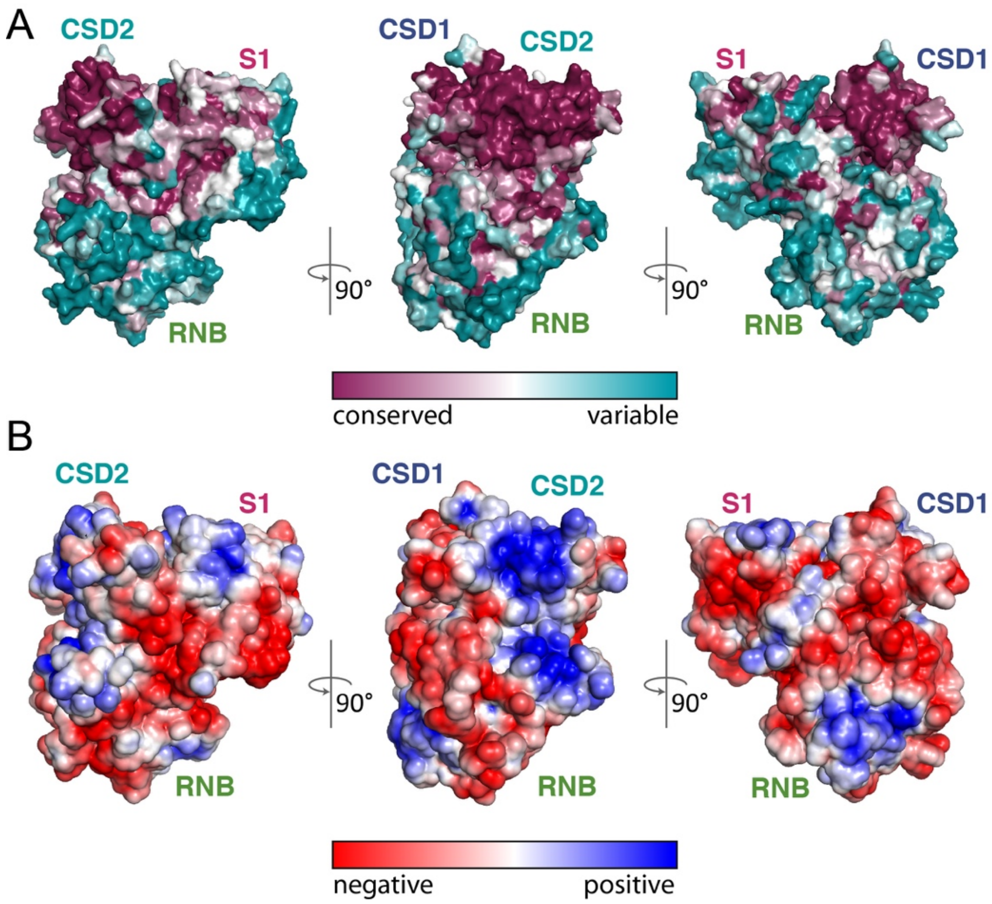

Figure S6. A conserved, positively-charged surface is a candidate RNA-binding site. (A) Ssd1 shown as a van der Waals surface coloured by conservation showing front, side and back views. Front (left) and side (middle) views match those in Figure 4B, while the left view is of the “back” of the molecule. Conservations scores were calculated using CONSURF. (B) The same views of Ssd1 showing surface electrostatics on a solvent accessible surface, calculated using APBS (www.poissonboltzmann.org). The gradient is from -3 to +3 kT/e.

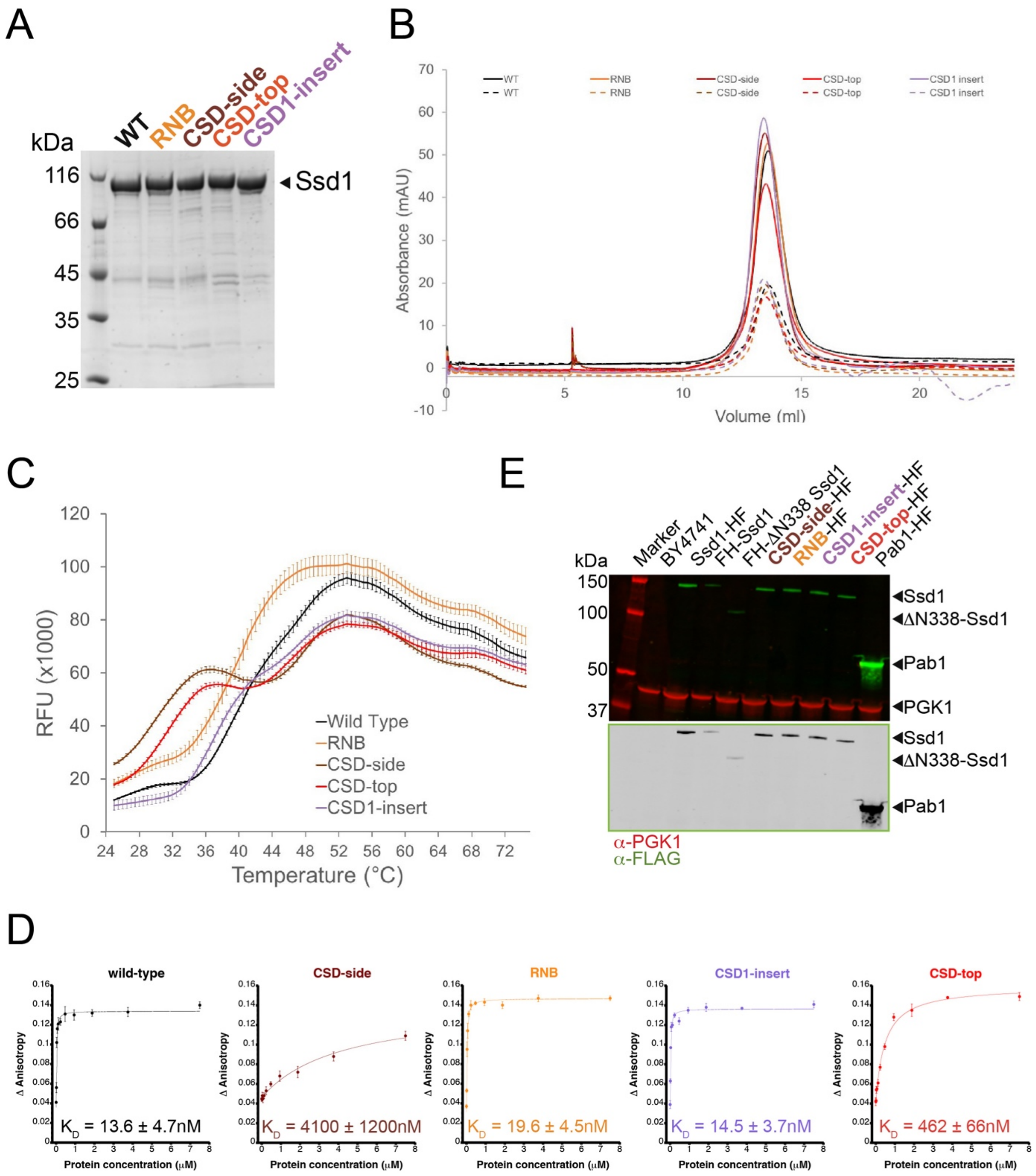

Figure S7 Mutant proteins are soluble and stable *in vitro* and expressed *in vivo*. (A) Samples of purified Ssd1 native and mutant sequences on Coomassie stained SDS-PAGE gel. (B) Analytical size exclusion chromatography profiles for each of the samples shown in (A). (C) Thermal denaturation profiles for each of the mutants shown in (A), showing fluorescence of Sypro Orange against temperature. (D) Raw data and fitted curves for fluorescence anisotropy data shown in Figure 6D. (E) Western blot of soluble protein extracted from yeast strains used for phenotyping assays. Rat α-FLAG (green) was used to probe tagged versions of Ssd1 and Pab1 and rabbit α-PGK1 (red) was used as a loading control. The lower greyscale panel shows data just from the green channel.

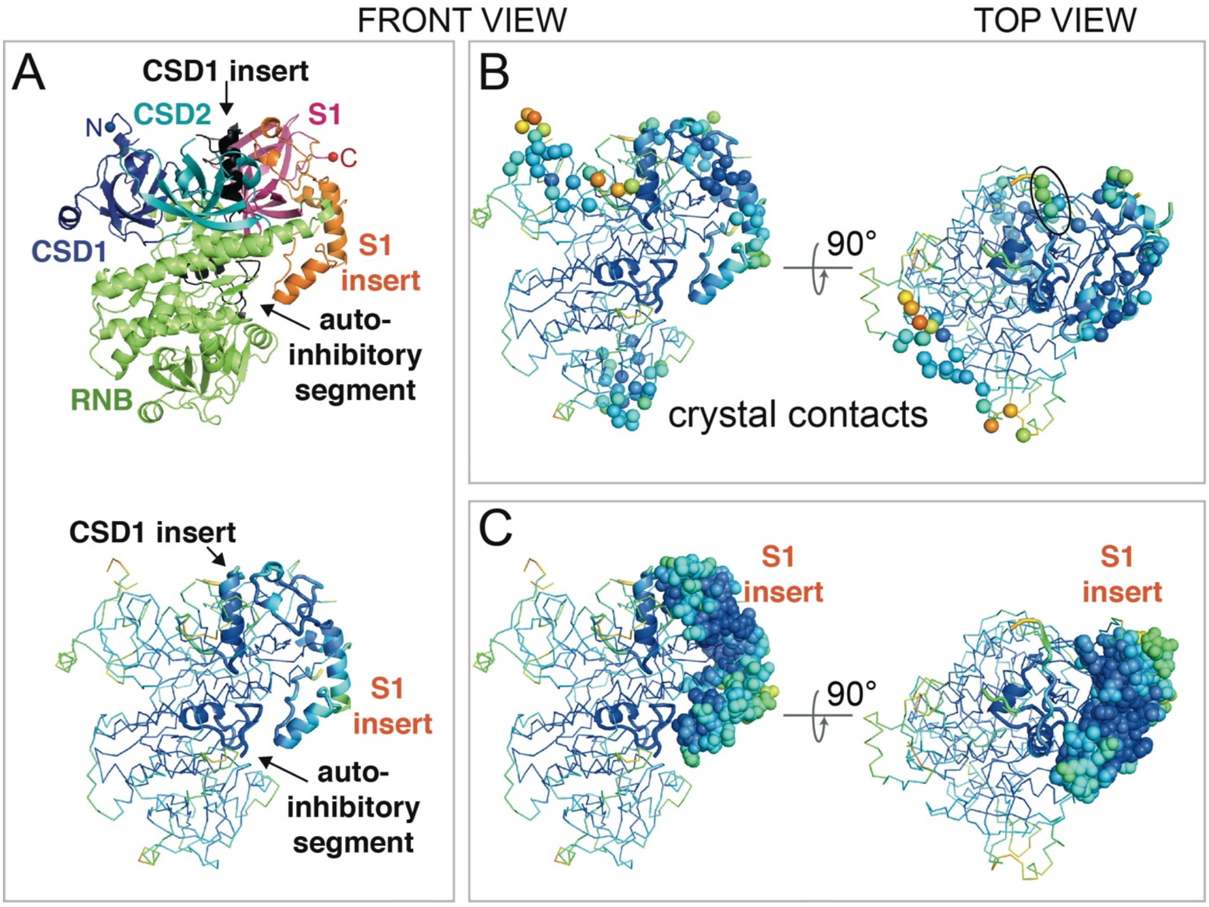

Figure S8 (**A**) The upper panel shows a typical representation of Ssd1 structure as cartoon highlighting the CSD1 insert and the autoinhibitory segment, both of which block the residual RNA binding and catalytic sites. The lower panel highlights these two elements as well as the S1 insertion as cartoons, while showing the rest of the structure as ribbons. The structure is coloured as a B-factor gradient from low (blue) to high (orange) B-factors. (**B**) A similar view to (A) is used, with an additional rotation around the x-axis to show the "top" view of Ssd1. Cα atoms of residues involved in crystal contacts are shown as spheres. The three residues of the CSD1 insertion that are involved in crystal contacts are indicated by the black oval. (**C**) The S1 insertion is shown as a space-fill model to highlight the interaction with the core domains of Ssd1. This interaction buries 2364 Å^2 of the surface of the core domains.

Supplementary methods

Protein extraction from yeast

Yeast strains were grown from overnight cultures of single colonies in 50ml of YPDA medium in 250ml conical flasks with a starting OD600 of 0.05 to 0.07, until they reached a final OD600 of 0.41-0.51. Final OD600 measurements were determined for normalisation. Samples were harvested by centrifugation at 2500x*g* for 3 minutes, the growth medium removed and the pellets washed in 10ml of sterile Phosphate Buffered Saline (PBS). Samples were centrifuged again before removing the PBS and resuspending the pellets in 1ml PBS. Samples were then transferred to 1.5ml in screwcap vials and centrifuging at 5000x*g* for 1 minute. Supernatants were removed and the samples were stored at 80°C. For extraction we followed the method of Kushnirov (1). Briefly, H_2O was added to resuspend the frozen yeast pellets. The volume was calculated as OD600*400µL. From the resuspended cells, 100 µL was transferred to a tube containing 100 µL of 0.2M NaOH and cells were incubated at room temperature for 5 mins. Cells were collected by centrifugation for 3.5 mins at room temperature at 16000 x g and resuspended in sample buffer (50 µL of 60 mM Tris-HCl pH6.8, 5% glycerol, 2% SDS 4% β-mercaptoethanol, 0.0025% bromophenol blue). Samples were boiled for 3 mins and cellular debris was collected by centrifugation for 5 min. The supernatant was transferred to a clean tube and a 1:5 dilution was made into sample buffer for analysis by SDS-PAGE.

Western blotting

Samples were run on 4%-20% gradient SDS-PAGE gel (BioRad) at 190 V for approximately 1 hour. The gel was then blotted on nitrocellulose membrane (0.2 µm, BioRad) using a wet transfer method for 2 hours and 20 mins at 100 mA. The blot was blocked in 5 % milk in PBS for 1 hour. Primary antibodies used were rabbit α-PGK1 (gift from Adele Marsden) used at 1/10 000 dilution and rat α-FLAG antibody (Agilent #200474-21) used at 1/2000 dilution, both in 5% milk, PBS +0.5% tween (PBS-T). After 1 hour, the membrane was washed three times in PBS-T for at least 5 mins. Secondary antibodies (IRDye 800CW α-rat, IRDye 680RD α-rabbit, both LICOR) were co-incubated on the blot for 1 hour at room temperature. These were used at 1/10 000 dilution in 3% BSA in PBS-T. After washing three time is PBS-T for at least 5 mins each, the blot was scanned using a LICOR Odyssey CLx scanner and images were analysed using Image Studio.

Semi-analytical Size Exclusion Chromatography

A Superdex 200 10/300 gel filtration column (Cytiva) was equilibrated with 20 mM HEPES pH 7.5, 150 mM NaCl, 1 mM DTT. 2 µM of wildtype and mutant Ssd1ΔN338 protein (native and mutants) were loaded into a 500 µl loop and injected onto the column one after the other. The absorbance values at 280 nm and 260 nm were recorded during their elution and the chromatograms were overlayed.

Thermal denaturation assay

Thermal denaturation experiments were carried out to study the stability of the purified Ssd1ΔN338 mutants. The reactions were assembled on ice in a 96-well PCR plate (BioRad) and performed as triplicates. 1 μM of protein was allowed to bind to 5X Sypro Orange (BioRad) in a reaction buffer consisting of 20 mM HEPES pH 7.5, 150 mM NaCl, 1 mM DTT. The 96-well plate was subjected to an incremental temperature gradient of 0.5°C ranging from 25 - 74.5°C in an iCycler iQ Real Time PCR machine (Biorad). The results were plotted as change in fluorescence units (RFU) against temperature gradient.

1. Kushnirov, V.V. (2000) Rapid and reliable protein extraction from yeast. *Yeast*, **16**, 857-860.
